# Quantitative limits of host-driven HIV transcription and host gene control by the viral transactivator Tat

**DOI:** 10.1093/nar/gkag631

**Published:** 2026-06-22

**Authors:** Chuan Li, Yuan Ma, Yi Wang, Huiming Yang, Susana T Valente

**Affiliations:** Department of Immunology and Microbiology, The Herbert Wertheim UF Scripps Institute for Biomedical Innovation & Technology, Jupiter, FL 33458, United States; Department of Immunology and Microbiology, The Herbert Wertheim UF Scripps Institute for Biomedical Innovation & Technology, Jupiter, FL 33458, United States; Department of Immunology and Microbiology, The Herbert Wertheim UF Scripps Institute for Biomedical Innovation & Technology, Jupiter, FL 33458, United States; Department of Immunology and Microbiology, The Herbert Wertheim UF Scripps Institute for Biomedical Innovation & Technology, Jupiter, FL 33458, United States; Department of Immunology and Microbiology, The Herbert Wertheim UF Scripps Institute for Biomedical Innovation & Technology, Jupiter, FL 33458, United States; Skaggs Graduate School of Chemical and Biological Sciences, The Scripps Research Institute, Jupiter, FL 33458, United States

## Abstract

HIV transcription is amplified by the viral transactivator Tat, yet two quantitative issues remain unresolved in chromatin: the extent to which host machinery sustains transcription in the absence of Tat, and why Tat’s widespread chromatin association yields limited host-gene output. We engineered an isogenic Tat-deficient derivative of the HIV_GKO_ dual-reporter virus that preserves native proviral architecture, enabling longitudinal measurements across diverse integration sites in primary CD4⁺ T cells and Jurkat cells. Across thousands of proviruses, host factors alone supported a restricted baseline, with Tat-null proviruses producing ∼4%–15% of wild-type protein output and remaining constrained under strong stimulation. Chromatin immunoprecipitation-seq profiling revealed that Tat is dispensable for promoter-proximal RNAPII engagement and pausing but required for efficient CDK9 recruitment, Ser2 phosphorylation, and productive elongation, defining a ceiling for Tat-independent transcription. Tat deficiency reduced overall RNAPII occupancy without increased promoter-proximal accumulation. Genome-wide mapping using Tat-null controls revealed broad Tat association with active host loci and modest increases in elongation-factor occupancy; however, host-gene RNA gains were small (∼1.05–1.49×) and poorly correlated with Tat binding. Instead, elongation-associated chromatin features better predicted responsiveness. Together, these findings define a framework separating host-driven transcription from Tat-dependent scaling and explain Tat’s disproportionate potency at the provirus.

## Graphical Abstract

**Graphical Abstract:**
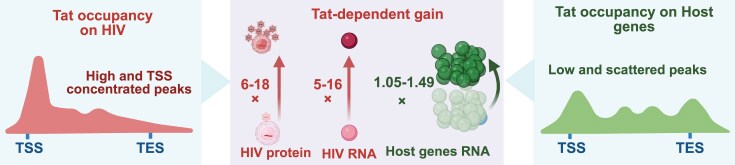

## Introduction

Integrated HIV-1 proviruses operate at the interface between host and viral transcriptional control, where layered regulatory mechanisms determine whether proviral genomes remain silent or become transcriptionally active. Host transcription factors and chromatin regulators establish the initial conditions for RNA polymerase II (RNAPII) recruitment and promoter activity at the viral 5′ long terminal repeat (5′LTR), whereas the viral transactivator Tat subsequently reprograms RNAPII dynamics to convert basal transcription into a highly processive and self-reinforcing transcriptional program. By binding the HIV transactivation response (TAR) RNA element, Tat recruits P-TEFb and the Super Elongation Complex, promotes RNAPII pause release, and drives robust transcriptional elongation [1–5]. Through additional interactions with histone acetyltransferases and chromatin-remodeling complexes, Tat reshapes nucleosome architecture at the LTR to further stabilize productive transcription [6–13]. Together, these mechanisms create a hierarchical coupling between host-driven initiation and Tat-dependent elongation, providing a molecular basis for the exceptional specificity, dynamic range, and magnitude of HIV transcriptional amplification [14–16].

Before Tat accumulation, HIV transcription is governed exclusively by host regulatory pathways. Cellular transcription factors such as NF-κB, NFAT, and AP-1, initiate low-level transcription at the viral promoter, generating the first Tat transcripts and enabling the transition to Tat-dependent elongation [17–20]. Although inhibition of Tat activity by small molecules or dominant-negative variants strongly suppresses viral reactivation, it fails to eliminate residual HIV RNA expression [21–24], revealing that Tat-independent transcription persists even under conditions of profound elongation blockade [25]. Despite decades of investigation, the quantitative magnitude and mechanistic basis of host-driven transcription from chromatin-integrated proviruses remain poorly defined, particularly in native genomic contexts where proviral chromatin architecture and integration-site diversity shape transcriptional potential.

Consequently, the physiological extent of Tat-independent HIV transcription has never been systemically quantified across diverse integration sites in native viral contexts. Most previous studies have relied largely on LTR-only reporter systems, which fail to recapitulate proviral chromatin organization and integration-site heterogeneity [26–29]. Consequently, the intrinsic capacity of host transcriptional machinery to drive proviral transcription, and the extent to which Tat selectively amplifies this activity relative to host genes, have remained unresolved. A fundamental quantitative question therefore persists: what is the maximal level of HIV transcription that host factors alone can sustain in a native chromatin context where Tat-TAR coupling is preserved?

Here, we address this gap by adapting a widely used dual-fluorescent HIV reporter construct, HIV_GKO_ [30–33], to generate an isogeneic Tat-null HIV_GKO_ reporter that preserves the native LTR and proviral genome while eliminating Tat expression. By integrating this platform with RNAPII-directed chromatin immunoprecipitation (ChIP)-seq and transcriptomic analysis, we quantatively define the physiological baseline of host-driven HIV transcription across thousands of independent integration sites and dissect the mechanistic contribution of Tat to transcriptional initiation and elongation. We further assess the extent to which Tat binding to host chromatin is associated with functional transcriptional regulation. Together, our results reveal that host factors alone support only a limited, quantitatively constrained level of HIV transcription and establish Tat as a selective and disporportionately potent amplifier of transcriptional elongation.

## Materials and methods

### Cell lines and cell culture

HEK293T cells (ATCC) were cultured in high-glucose Dulbecco’s modified Eagle’s medium (Gibco) supplemented with 10% heat-inactivated fetal bovine serum (FBS; Atlas Biologicals, #FS-0500-AD) and penicillin-streptomycin-glutamine (PSG; penicillin 100 U/ml, streptomycin 100 µg/ml, L-glutamine 2 mM; Thermo Fisher, #10378016). Jurkat CD4⁺ T cells and derived HIV-integrated populations were maintained in RPMI 1640 (Thermo Fisher, #11875093) supplemented with 10% FBS and PSG. Cells were routinely tested for mycoplasmas contamination and maintained at 37°C in a humidifier incubator with 5% CO_2_.

Primary naïve CD4⁺ T cells were isolated from HIV-1-negative donors using magnetic negative selection (Naïve CD4⁺ T Cell Isolation Kit II, Miltenyi Biotec, #130-094-131). Cells were differentiated into central memory-like CD4⁺ T cells as previously described [34, 35]. Briefly, cells were activated with anti-CD3/CD28 Dynabeads (Thermo Fisher, #11131D, 1:1 cell-to-bead ratio) in the presence of anti-IL-4, anti-IL-12, and TGF-β1 [1 µg /ml, 2 µg/ml, and 10 ng /ml, respectively (Peprotech)]. After bead removal, cells were cultured in RPMI supplemented with IL-2 (30 IU/ml).

### HIV_GKO_ constructs and viral production

The HIV_GKO_ dual-reporter virus (Addgene, #112234) was used as the wild-type (WT) backbone. Tat-deficient derivatives were generated by site-directed mutagenesis. The TatLeak mutant was generated by substituting the Tat start site codon (ATG to ACG) to reduce Tat translation without disrupting overlapping Vpr’s open reading frames. The TatNull construct was generated by introducing two premature stop codons downstream of the Tat coding sequence while preserving Vpr coding potential. All constructs verified by Sanger sequencing to confirm precise mutagenesis.

VSV-G-pseudotyped HIV_GKO_ constructs were produced in HEK293T cells by co-transfection of HIV_GKO_ plasmids and pCMV-VSV-G (Addgene, #8454) using TransIT®-2020 Transfection Reagent (Mirus Bio, #MIR 5404). Viral supernatants were collected at 48–72 h, filtered (0.45 µM), and virus titers determined by infection in Jurkat CD4^+^ T cells for 3 days.

### Luciferase reporter assay to assess residual Tat activity

HeLa cells stably expressing an LTR-luciferase reporter (HeLa-LTR-Luc) were seeded in 12-well plates and transfected with HIV_GKO_ constructs or control plasmid mKO2-N1 (Addgene #54625) using TransIT-LT1 (Mirus #MIR2306) according to the manufacturer’s instructions. Transfection efficiency was monitored by mKO2 fluorescence and used for normalization of luciferase signals. Cells were harvested 48 h post-transfection and lysed in 1× passive lysis buffer (Promega, #E194A). Luciferase activity was quantified using the Luciferase Assay System (Promega, #E1501) on a Berthold luminometer and normalized to total protein content determined by Bradford assay (Bio-Rad #5000006). Data are presented as relative light units. All experiments were performed in triplicate.

### Viral infection and generation of integrated populations

Jurkat cells were infected with TatWT, TatLeak, and TatNull at multiplicity of infection (MOI) of ∼0.2, a low-MOI condition used to enrich for single proviral integration and preserve integration-site heterogeneity across the population. Integrated populations were generated by sorting mKO2^+^ cells using flow cytometry (BD FACSAria Fusion). For primary CD4⁺ T cells, infection was performed on day 7 of differentiation by spinoculation (1740 × *g*, 2 h, 37°C) using 500 ng p24 per 10^6^ cells. Infected cells were analyzed every 3 days by flow cytometry.

### Cellular stimulation and pharmacological treatments

Cells were stimulated with phorbol 12-myristate 13-acetate [PMA 10 nM (Fisher, #BP6851)]. Trichostatin A [TSA 2 µM (Sigma, #T-1952)], TNF-α (20 ng/ml, Cell Signaling Technology, #8902SC), or JQ1 (10 µM, MedchemExpress, # HY-13030) for the indicated times. Cell counts and viability were determined using trypan blue exclusion and a Cellometer Auto T4 (Nexcelom).

### Flow cytometry

Primary T cell activation was assessed by surface staining for CD25 (BD Biosciences, #560503) and CD69 (BD Biosciences, #561928). Cell cycle analysis was performed by fixation followed by staining with propidium iodide (PI) using FxCycle™ PI staining solution (Molecular probes, #F10797) and quantification of DNA content by flow cytometry. Intracellular HIV p24 staining was performed using an APC-conjugated anti-p24 antibody (MediMabs, MM-0289) following fixation with formaldehyde-containing buffer (BD Biosciences, 51-2090KZ) and permeabilization in Perm/Wash Buffer (BD Biosciences, 554723#). Data acquisition was performed on BD LSRFortessa or FACSCanto instruments. In Jurkat T cells, GFP expression was used as a readout of HIV transcription, while mKO2 expression was used to identify cells harboring integrated proviruses. In primary CD4^+^ T cells, infected (integrated) cells were defined as GFP⁺ or mKO2⁺; as previously described [31]. Mean fluorescence intensity (MFI) and frequencies of GFP⁺ cells within the mKO2⁺ population were quantified. Data were analyzed using FlowJo v10, and statistical analyses were performed using GraphPad Prism.

### Western blotting

Cells were lysed in RIPA buffer supplemented with protease inhibitors (Roche, #04293132001). Protein concentrations were determined by Bradford assay. Equal amounts of protein were resolved by sodium dodecyl sulfate–polyacrylamide gel electrophoresis and transferred to PVDF membranes. Membranes were probed with antibodies against Tat (Thermo Fisher, #MA1-71509), p55 Gag (BEI resources, #ARP-13417), and HDAC1 as loading control (Thermo Fisher, #PA1-860), followed by HRP-conjugated secondary antibodies. Signals were detected using ECL^TM^ Prime Luminol Enhancer solutions (Cytiva) and quantified using Image Lab software (Bio-Rad Laboratories).

### RNA isolation and quantitative reverse transcriptase-polymerase chain reaction

Total RNA was extracted using RNeasy kit (Qiagen, #74106) and treated with DNase (Invitrogen, #AM1907). Complementary DNA was synthesized using random hexamer and SuperScript III First Strand Synthesis kit (Invitrogen, #18080051). Quantitative polymerase chain reaction (PCR) was performed using SensiFAST^TM^ SYBR® No-ROX Kit (BioLine, #BIO-98020). HIV transcripts were normalized to RPL13A expression (Table 1) using the ΔΔCt method.

**Table 1.** Primers used for quantitative RT-PCR

Target	Forward oligos	Reverse oligos
Total HIV mRNA	GGTTAGACCAGATCTGAGCCTGG	CAACAGACGGGCACACACTACT
Gag mRNA	GCAATGAGCCAAGTAACAAATCCA	CCTTTTTCCTAGGGGCCCTGC
Tat/Rev mRNA	TCTATCAAAGCAACCCACCTC	CGTCCCAGATAAGTGCTAAGG
RPL13A	GCCCTACGACAAGAAAAAGCG	TACTTCCAGCCAACCTCGTGA

### Chromatin immunoprecipitation and library preparation

ChIP was performed as previously described [36]. Cells were crosslinked with 1% formaldehyde for 10 min and quenched with 0.125 M glycine. Chromatin was sonicated to 200-400 bp fragments (Qsonica, #Q800R3 sonicator). A total of 20 μg chromatin was used for 2 μg antibody of Tat (Thermo Fisher, #MA1-71509), CDK9 (Protintech, #11705-1-AP), RNAPII (Active Motif #61801), S2P-RNAPII (Active Motif, #61803), S5P-RNAPII (Active Motif, #61805), or matched IgG controls, mouse IgG (Millipore # NI03), rabbit IgG (Fisher Scientific, #02-6102), and rat IgG (Thermo Fisher, #31933). The equivalent of 5% chromatin was saved as input. DNA was purified, treated with RNase A (Thermo Scientific, #FEREN0531), reverse crosslinked, and then digested with proteinase K (Fisher Scientific # BP1700100). The DNA was purified using a PCR clean kit (Qiagen, #28106).

ChIP enrichment was validated by qPCR prior to sequencing as previously described [35, 37]. ChIP DNA libraries were prepared using the NEB Next Ultra II DNA Library Prep Kit (NEB, #E7103) and indexed using unique dual indices. Libraries were sequenced on an Illumina platform with paired-end reads. Unless otherwise stated, ChIP-seq experiments were performed with two independent biological replicates per condition.

### ChIP-seq data processing, normalization, and peak calling

Sequencing quality was assessed using FastQC and MultiQC. Reads were trimmed using Trim Galore and aligned to a custom GRCh38 reference genome containing the HIV_GKO_ provirus as an additional chromosome using Bowtie2 (very-sensitive preset and a maximum fragment length of 2000 bp). Alignments were sorted/indexed with santools; duplicate reads were removed using Picard. Genome-wide signal tracks were generated using deepTools bamCoverage with RPGC normalization to enable quantitative comparison across samples, using hg38 effective genome size (2.91 × 10^9^) with 10 bp bins and read extension while ignoring marked duplicates.

Peak calling was performed using MACS2 in paired-end-mode (-f BAMPE) with narrow peak detection and a false discovery rate (FDR) threshold of *q* < 0.05 using matched input controls. Overlap analysis were performed using bedtool intersect; enrichment against randomized background was assed using bedtools shuffle excluding blacklist regions.

TatNull-corrected Tat occupancy. To account for nonspecific background and transcription-coupled association, Tat ChIP-seq analyses at host genes used TatNull as an empirical background. For metagene and gene-level analyses, TatWT RPGC signal was corrected by subtracting the corresponding TatNull RPGC signal on a per-region basis prior to aggregation (referred to as “TatNull-corrected” Tat signal).

### Tat chromatin occupancy classification

Tat-bound genes were classified as Tat^−^, Tat^+^, or Tat^++^ based on Tat ChIP-seq peak overlap and enrichment.

### Metagene profiling and gene-level ChIP-seq quantification

For metagene analysis, signal matrices were computed using deepTools computeMatris in scale-region mode with 6 kb upstream, a scale gene body, and 6 kb downstream using 25 bp bins. PCGs and lncRNAs were analyzed seperately and subdivided into Tat^−^, Tat^+^, and Tat^++^ classes.

Gene-level ChIP-seq signal was quantified using deepTool multiBigwigSummary on preferred promoter and gene-body windows. Promoter regions were defined as TSS-50 to TSS+ 350 nt, and gene bodies were defined as TSS + 350 nt to transcription end site (TES), thereby excluding promoter-proximal pausing regions from gene-body measurements. Violin plots and summary statistics were generated using ggplot2. Statistical significance was assessed using two-sided Wilcoxon rank-sum tests with multiple-testing correction where indicated.

### Initiation and elongation indices at the HIV provirus

The initiation index was calculated as the ratio of S5P to total RNAPII signal at the HIV promoter (TSS-50 to TSS+350 nt) [38, 39]. The elongation index (EI) was calculated as S2P signal across the HIV gene body (TSS+350 nt to TES) normalized to the promoter RNAPII occupancy [40–42]. Indices were computed from RPGC-normalized signals and averaged across biological replicates.

### RNA-seq processing, quantification, and differential expression analysis

Total RNA libraries were prepared using the NEBNext Ultra II Directional RNA Library Prep Kit and sequenced on an Illumina platform. Raw paired-end RNA-seq reads were assessed using FastQC and summarized with MultiQC. Adapter trimming and quality filtering were performed using Cutadapt. Trimmed reads were quantified using Salmon in quasi-mapping mode against a combined transcriptome (GENCODE v44 human transcripts supplemented with HIV_GKO_ transcripts). Transcript abundances were summarized to gene-level counts using tximport with lengthScaledTPM.

Gene-level counts were analyzed using DESeq2. Size factors were estimated using the median-of-ratios method; dispersions were fitted using the default model. Differential expression testing was performed using Wald tests with Benjamini–Hochberg FDR correction.

### Tat-dependent stimulation metrics and integrative analyses

Δ-signal (TatWT_stim_ versus TatNull_stim_). For Fig. 4 and related analysis, Δ-signal was defined as log_2_ (TatWT_Stim_ / TatNull_Stim_).

ΔΔ (interaction-based Tat-dependent stimulation gain). To quantify Tat-dependent stimulation gain, a DESeq2 intersection framework was used with genotypes (TatWT versus TatNull), stimulation (Stim versus NS), and their interaction. For each gene, Tat-dependent stimulation gain (ΔΔ) was defined as: ΔΔRNA = (TatWT_Stim_ − TatWT_NS_) − (TatNull_Stim_ − TatNull_NS_), using log_2_ (normalized counts + 1). This interaction-based framework isolates the Tat-specific component of the stimulation response by controlling for basal expression differences and Tat-independent stimulation effects. ΔΔRNA values were used for gene-level and population-level analyses. The HIV provirus was excluded from host-gene statistical testing and shown only as a scale reference where indicated. Genes with a significant interaction term (adjusted *P* < .05 and |log_2_ fold change| ≥ 0.5) were defined as Tat-dependent stimulation-response genes for downstream analyses.

Quartile-based intersection analysis (UpSet). For Fig. 6D, Tat-bound genes were ranked independently by ΔΔRNA, ΔΔTat gene-body occupancy, and ΔΔS2P gene-body occupancy, and the top quartile of each metric was selected. Intersections were computed to identify genes exhibiting coordinated Tat-dependent changes in Tat occupancy, elongation-associated chromatin features, and RNA output.

### Functional enrichment analysis

Gene Ontology enrichment analyses were performed using clusterProfiler (enrichGO). Background gene sets were defined as all annotated gene in org.Hs.eg.db. Adjusted *P*-values were calculated using the Benjamini–Hochberg method.

### Statistical analysis

Unless otherwise stated, data are presented as mean ± s.e.m. Pairwise comparisons were performed using two-sided tests as appropriate [including Wilcoxon rank-sum tests for distribution comparisons and analysis of variance (ANOVA) where indicated]. Multiple-testing correction was applied using Benjamini–Hochberg methods where appropriate. All plots were generated using ggplot2 and custom R scripts. Sample sizes, replicates, and statistical tests are indicated in figure legends.

## Results

### Tat-independent transcription is quantitatively constrained across primary and CD4^+^ T-transformed cells

To quantify host-driven HIV transcription in the absence of Tat, we engineered an isogeneic Tat-deficient derivative of the HIV_GKO_ dual-reporter virus [31] (Supplementary Fig. S1A). In this system, the HIV promoter (5′LTR) drives GFP expression as a readout of proviral transcription, while a constitutive EF1α promoter drives mKO2 to mark cells harboring integrated proviruses independent of transcriptional activity (Supplementary Fig. S1A). To progressively disrupt Tat expression, we generated two Tat mutant viruses. Mutation of the Tat start codon (ATG to ACG; TatLeak) reduced Tat translation without perturbing overlapping viral reading frames (Vpr), whereas introduction of premature stop codons downstream of the Tat coding sequence generated a true Tat-null virus (TatNull), without altering Vpr coding potential (Supplementary Fig. S1A). In HeLa-LTR-luciferase reporter assays [36], TatLeak retained partial Tat transactivation activity, whereas TatNull exhibited no detectable Tat activity (Supplementary Fig. S1B), confirming a complete ablation.

**Figure 1.**
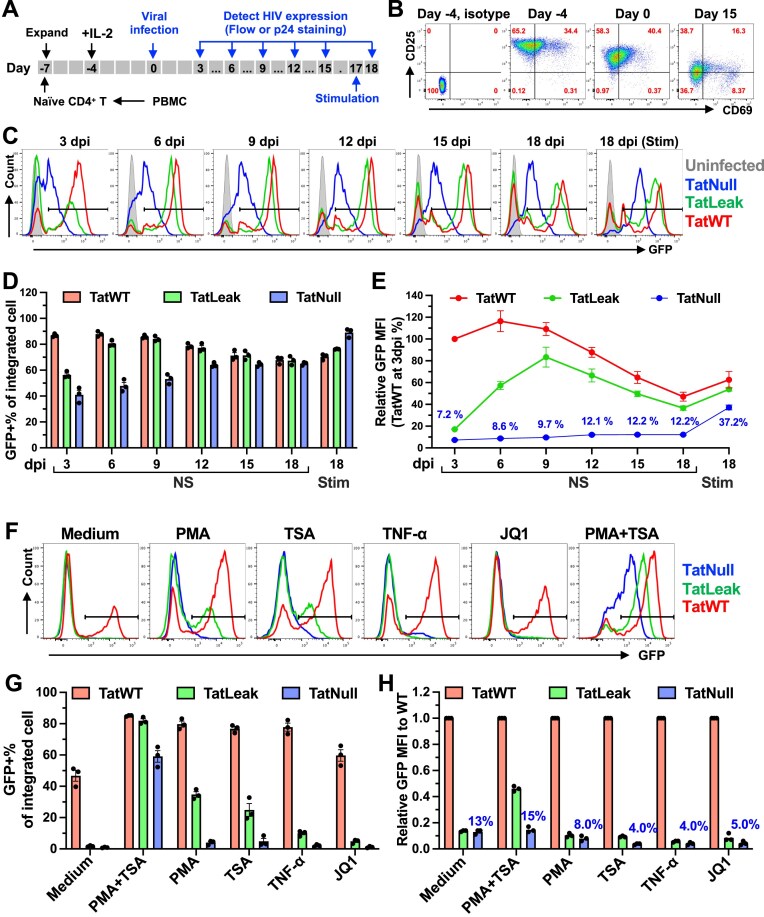
Tat-independent HIV transcription is quantitatively constrained in primary CD4^+^ T and Jurkat cells. (**A**) Schematic of experimental workflow for activation, infection with TatWT, TatLeak, or TatNull HIV_GKO_ viruses, and longitudinal monitoring of HIV expression. (**B**) Flow cytometry analysis of activation markers in uninfected cells. Representative CD25 versus CD69 staining at indicated time points (day −4 and day 0 relative to infection, and day 15 post-infection), with corresponding isotype controls. (**C**) Representative GFP fluorescence profiles of infected primary central memory CD4⁺ T cells at indicated days post-infection (dpi). GFP expression was analyzed within the integrated (mKO2⁺) population. Quantification of proviral transcription in primary T cells measured as the fraction of GFP⁺ cells (**D**) and GFP MFI normalized to TatWT at 3 dpi (**E**) across time points under non-stimulated (NS) and stimulated (10 nM PMA + 2 µM TSA) conditions. (**F**) Representative GFP histograms of Jurkat cells infected with TatWT, TatLeak, or TatNull HIV_GKO_ viruses following treatment with indicated latency-reversing agents. On 4 dpi, cells were treated overnight with 10 nM PMA, 2 µM TSA, 20 ng/ml TNF, 10 µM JQ1, or the combinantion 10 nM PMA + 2 µM TSA. Quantification of GFP⁺ frequency (**G**) and GFP MFI normalized to WT (**H**) in Jurkat cells under indicated stimulation conditions (16 h on 4 dpi). Data in panels (D–G) represent mean ± s.e.m. with individual biological replicates shown as points.

To investigate Tat-dependent transcription in a physiologically relevant context, we employed a widely used primary CD4⁺ T-cell infection model in which naïve cells are activated and expanded prior to infection [34, 43], followed by longitudinal monitoring of viral expression (Fig. 1A). Flow cytometric analysis of activation markers confirmed that cells remained in an activated or transitioning state at the time of infection (Fig. 1B), consistent with a transcriptionally permissive chromatin environment. Given prior reports that the constitutive EF1α promoter can be partially silenced in primary T cells [31, 44], we directly compared GFP-based detection of HIV expression with intracellular p24 staining. At 3 dpi, cells infected with TatWT, TatLeak, and TatNull HIV_GKO_ viruses were analyzed by flow cytometry using GFP, p24, and mKO2 signals (Supplementary Fig. S1C). Within the integrated cell population, defined as GFP⁺ or mKO2⁺ (and independently as p24^+^ or mKO2^+^) [31], GFP and p24 measurements were highly concordant, both in the fraction of expressing cells (GFP⁺ versus p24⁺) and in relative MFI (Supplementary Fig. S1C and D). Together, these results validate GFP as a robust and quantitative reporter for HIV gene expression in this system.

Using this framework, TatWT, TatLeak, and TatNull HIV_GKO_ viruses exhibited comparable integration efficiencies in primary central memory CD4⁺ T cells, as indicated by similar frequencies of integrated cells at early time points (Supplementary Fig. S1E and F), thereby excluding differential infection as a confounding factor. Proviral transcription was therefore quantified by GFP expression within the integrated population (Fig. 1C). Over time, however, the fraction of integrated cells declined more rapidly in TatWT infections compared to TatNull, consistent with a selective loss of cells expressing higher levels of viral gene products (Supplementary Fig. S1F). In line with this, TatWT proviruses rapidly reached high GFP expression, whereas the TatLeak and TatNull proviruses exhibited progressively reduced transcriptional output (Fig. 1C and D). TatLeak proviruses showed an early defect that partially recovered toward WT levels over time. In contrast, TatNull proviruses displayed a sustained reduction in transcriptional output despite a gradual increase in the proportion of of GFP^+^ cells (Fig. 1C and D).

To correct for the time-dependent loss of highly expressing TatWT cells, GFP MFI values were normalized to TatWT at 3 dpi (Fig. 1E). Using this approach, TatNull proviruses exhibited consistently low transcriptional output, reaching only ∼7%–12% of TatWT levels across multiple time points (Fig. 1D). These results define a substantial, yet quantitatively constrained, Tat-independent transcription output driven by host factors alone, demonstrating that Tat is dispensable for proviral integration and basal transcriptional engagement but essential for robust transcriptional amplification. The distinct phenotypes of TatLeak and TatNull viruses further demonstrate that even minimal Tat expression is sufficient to restore near-WT transcriptional output, whereas complete Tat loss reveals a stable quantitative limit of host-driven transcription.

To determine whether strong activation can compensate for Tat loss, infected primary CD4^+^ T cells were stimulated with PMA and TSA at 17 days post infection (dpi). Consistent with a pre-activated primary T-cell state, stimulation only modestly increased gene expression from TatWT proviruses, but markedly enhanced gene transcription from TatNull proviruses, with GFP MFI increasing ∼3-fold. When normalized to TatWT at 3 dpi, this corresponded to ∼37% of TatWT levels (Fig. 1C–E). Despite this increase, TatNull expression remained substantially below TatWT, even under strong activating conditions, revealing a quantitative ceiling for Tat-independent transcription.

We next examined Tat-independent transcription in Jurkat T cells using low-MOI infections (Supplementary Fig. S1G). Under basal conditions, TatNull proviruses exhibited minimal GFP expression, whereas TatWT were transcriptionally active (Fig. 1E and F). Single latency-reversing agents induced modest activation of TatWT and TatLeak proviruses but had little effect on TatNull proviruses. Strikingly, combined PMA and TSA stimulation activated a large fraction of TatNull-infected cells, increasing the proportion of GFP^+^ to >60%; however, GFP MFI remained limited to ∼15% of TatWT levels, comparable to the ∼13% observed under basal conditions (Fig. 1F and G). These findings demonstrate that strong upstream signaling can initiate transcription in the absence of Tat but cannot substitute for Tat-dependent transcriptional amplification.

Together, these results establish that host transcriptional machinery can engage the HIV promoter and initiate transcription, but that Tat-independent transcription is quantitatively constrained to ~4%–15% of WT output across cell types. The TatLeak intermediate phenotype further underscores the exquisite sensitivity of HIV transcriptional output to Tat dosage and validates TatNull as a stringent reference for defining the physiological baseline of host-driven proviral transcription.

### Tat is required for efficient CDK9 and RNAPII recruitment and productive HIV elongation

To define the contribution of Tat to proviral transcription, we generated stable Jurkat cell populations harboring either TatWT or TatNull proviruses (Supplementary Fig. S2A). GFP analysis under NS and PMA + TSA-stimulated (Stim) conditions confirmed markedly reduced transcriptional activity in TatNull cells (Supplementary Fig. S2A). Consistently, total HIV and Gag transcripts were substantially reduced in TatNull cells (∼6% of TatWT under NS and ∼11% upon stimulation), while Tat/Rev transcripts showed a similar reduction at basaline (∼6%) but increased to ∼30% of TatWT upon stimulation (Supplementary Fig. S2B), suggesting partial recovery of spliced transcripts under strong activation.

Western blot analysis confirmed the absence of Tat and reduced Gag expression in TatNull cells (Supplementary Fig. S2C), and Sanger sequencing verified the introduced mutations (Supplementary Fig. S2D). Cell cycle analysis by propidium iodine (PI) staining showed no major differences between TatWT and TatNull populations (Supplementary Fig. S2E), indicating that the observed effects are not attributable to altered cell cycle distribution.

**Figure 2.**
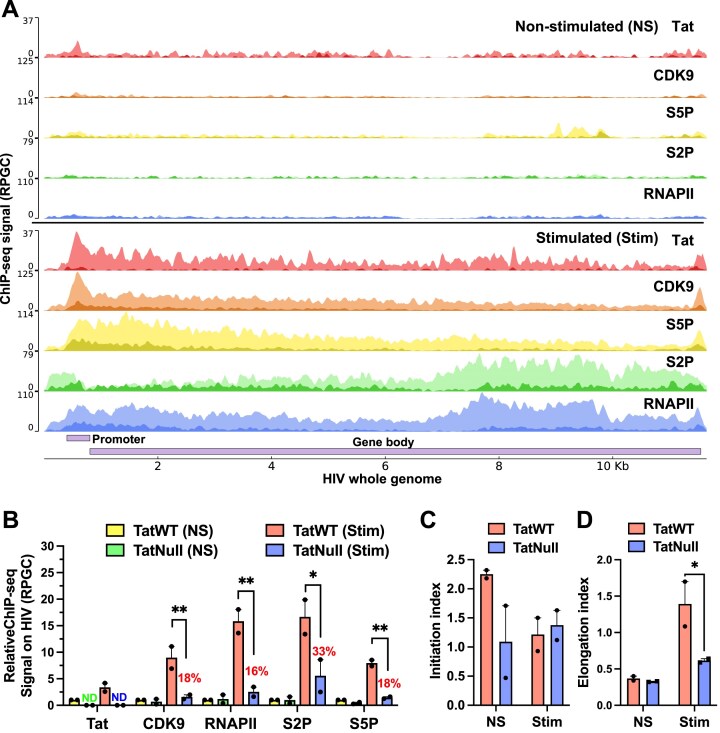
Tat is required for efficient CDK9 recruitment and productive HIV transcriptional elongation. (**A**) Genome browser tracks showing RPGC-normalized ChIP-seq signal for Tat, CDK9, total RNAPII, RNAPII-Ser5P (S5P), and RNAPII-Ser2P (S2P) across the integrated HIVGKO provirus in TatWT and TatNull Jurkat cells under NS and stimulated (PMA + TSA) conditions (TatWT: light color, TatNull: dark color). (**B**) Quantification of mean ChIP-seq signal across the HIV_GKO_ provirus for each factor in TatWT and TatNull cells under NS and stimulated conditions, normalized to WT NS. Percent values indicate TatNull signal as a fraction of TatWT under stimulated conditions; ND, not detected. Points indicate biological replicates. (**C, D**) Initiation and elongation indices at the HIV promoter and gene body, respectively (defined in “Materials and methods” section), quantifying promoter engagement and productive elongation. Data in panels (B–D) are shown as mean ± s.e.m. Statistical significance is indicated in the figure and assessed using two-way ANOVA; *P < .05, **P < .01.

We next performed ChIP-seq profiling of Tat, CDK9, total RNAPII, RNAPII-Ser5P (S5P), and RNAPII-Ser2P (S2P) under basal and PMA + TSA-stimulated conditions. Genome browser visualization revealed minimal basal occupancy of Tat, CDK9, and RNAPII across the provirus in both genotypes (Fig. 2A). Upon stimulation, WT proviruses exhibited robust recruitment of Tat, CDK9, RNAPII, S2P, and S5P across both promoter and gene body, with Tat enrichment extending beyond the TAR region and closely paralleling elongation-associated factors. In contrast, TatNull proviruses displayed stimulus-induced recruitment of RNAPII and CDK9, but at substantially lower magnitude and with reduced gene-body occupancy (Fig. 2A). Consistent with this, both total RNAPII and RNAPII-Ser5P signals were globally reduced in TatNull proviruses, indicating diminished overall polymerase engagement.

Quantitative analysis of RPGC-normalized ChIP-seq signal across the provirus confirmed these patterns (Fig. 2B). Stimulation markedly increased CDK9, total RNAPII, S5P, and S2P occupancy on TatWT proviruses, whereas TatNull proviruses exhibited consistently attenuated recruitment of all marks. This reduction extended to promoter-proximal RNAPII and S5P signals, indicating reduced polymerase engagement at the promoter in the absence of Tat, although direct assessment of pre-initiation complex (PIC) assembly was not performed. Under stimulated conditions, total RNAPII occupancy on TatNull proviruses reached ∼16% of TatWT levels, closely mirroring the residual transcriptional output observed in Fig. 1.

To dissect distinct transcriptional phases, we calculated initiation and elongation indices (Fig. 2C and D). The initiation index, defined as promoter-proximal S5P relative to total RNAPII [38, 39], was comparable between TatWT and TatNull proviruses and showed minimal change upon stimulation. In TatWT proviruses, the modest decrease in initiation index following stimulation likely reflects redistribution of RNAPII from the promoter into the gene body associated with enhanced elongation, rather than reduced initiation. These data indicate that Tat is largely dispensable for promoter-proximal RNAPII and paused polymerase distribution, despite reduced overall RNAPII levels. In contrast, the EI, defined as gene-body S2P relative to promoter RNAPII [40–42], increased ∼3.8-fold in TatWT proviruses but only ∼1.8-fold in TatNull proviruses following stimulation. Thus, although TatNull proviruses retain the capacity for RNAPII recruitment and transcription initiation, they exhibit reduced RNAPII occupancy and a marked defect in P-TEFb-dependent pause release and productive elongation.

Together, these data demonstrate that Tat is not required for establishing promoter-proximal RNAPII engagement but enhances overall RNAPII occupancy and is essential for efficient CDK9-dependent productive elongation, providing a mechanistic basis for the quantitative ceiling of Tat-independent HIV transcription.

### Tat associates broadly with host chromatin but is quantitatively enriched at a restricted gene subset

Although Tat is classically viewed as a provirus-specific transcriptional regulator, we asked whether Tat also associates with host chromatin on a genome-wide scale. To identify bona fide Tat-binding sites, TatNull samples were used as a background control. Replicate correlation analysis revealed clear separation between TatWT and TatNull ChIP-seq profiles, with increased concordance after stimulation and tight clustering of TatNull samples at background levels (Supplementary Fig. S3A). Using this TatNull-controlled framework, Tat peaks were independently called in TatWT and TatWT + Stim samples, identifying 1468 high-confidence Tat binding sites genome-wide in the stimulated TatWT condition.

Tat binding co-localized extensively with transcriptional machinery. Between 40% and 65% of Tat peaks overlapped CDK9, total RNAPII, S5P, or S2P, representing >100-fold enrichment relative to shuffled genomic controls (Supplementary Fig. S3B). Tat peak intensity correlated most strongly with CDK9 and total RNAPII occupancy, and more modestly with RNAPII phosphorylation states (Supplementary Fig. S3C), consistent with Tat engagement at transcriptionally active loci and its established interaction with P-TEFb.

**Figure 3.**
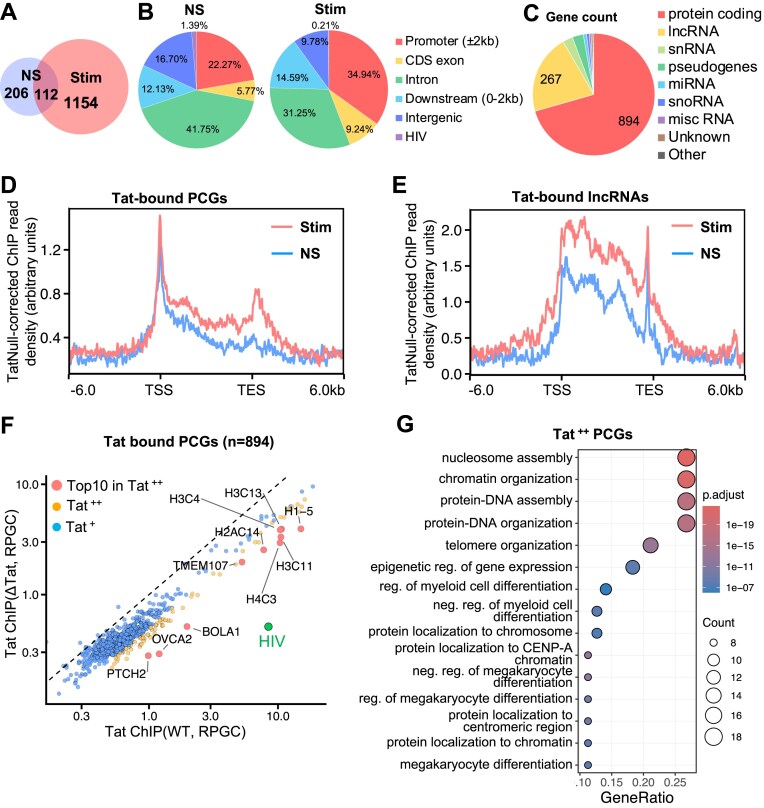
Genome-wide mapping identifies Tat-bound host genes and a highly Tat-enriched subset. (**A**) Overlap of Tat-bound genes detected in unstimulated (NS) and PMA + TSA-stimulated (Stim) TatWT cells. (**B**) Genomic distribution of Tat ChIP-seq peaks across promoters, exons, introns, downstream regions, intergenic regions, and the HIV provirus under NS and Stim conditions. (**C**) Biotype composition of Tat-bound host genes (*n* = 1267), including PCGs (*n* = 894) and long noncoding RNAs (lncRNAs, *n* = 267). (**D, E**) Metagene profiles of TatNull-corrected, RPGC-normalized Tat ChIP-seq signal across Tat-bound PCGs (D, *n* = 894) and lncRNAs (E, *n* = 267) under NS and Stim conditions. Signal is averaged across scaled gene bodies with ±6 kb flanking regions. (**F**) Gene-level comparison of Tat ChIP-seq signal between TatWT and TatNull cells across Tat-bound PCGs. Tat^+^ (*n* = 894), Tat^++^ (*n* = 81), and representative Tat^++^ genes are indicated; the HIV provirus is shown as a reference point (green). (**G**) Gene Ontology enrichment of Tat^++^ PCGs (*n* = 81). Dot size indicates gene counts and color indicates Benjamini–Hochberg-adjusted *P* values.

Consistent with Tat’s preferential association with active transcription complexes [45, 46], increased Tat abundance upon stimulation dramatically expanded the repertoire of Tat-bound genes, increasing from 318 loci at baseline to 1267 after stimulation, with 112 shared between conditions (Fig. 3A). Tat peaks were distributed across promoters, introns, and downstream regions, and stimulation increased Tat occupancy proportionally across genomic features rather than redistributing binding to specific regions (Fig. 3B). Among stimulated Tat-bound genes, PCGs (*n* = 894) predominated, followed by long noncoding RNAs (lncRNAs; *n* = 267) (Fig. 3C).

Metagene analysis (TatNull-corrected, RPGC-normalized signal) revealed a characteristic bimodal Tat distribution at PCGs, with enrichment at both transcription start sites (TSSs) and TESs, which was enhanced under increased Tat abundance after stimulation and accompanied by increased gene-body signal (Fig. 3D). Tat-bound lncRNAs exhibited broader promoter-proximal enrichment with similar Tat abundance-dependent increases (Fig. 3E). Thus, increased Tat availability primarily increased the magnitude of Tat association rather than altering its positional pattern along genes.

Functional annotation of Tat-bound PCGs revealed strong enrichment for chromatin-associated processes, including chromatin organization, nucleosome assembly, and epigenetic regulation (Supplementary Fig. S4A). Tat-bound lncRNAs displayed more limited functional enrichment, largely restricted to RNA metabolic and ribonucleoprotein-associated processes (Supplementary Fig. S4B), consistent with heterogeneous lncRNA function and incomplete annotation. Stratification by Tat signal intensity uncovered a distinct subset of genes with disproportionately high Tat occupancy (Tat^++^; *n* = 81; Supplementary Table S1) , exhibiting ≥2-fold higher Tat binding in TatWT + Stim compared to TatNull + Stim (Fig. 3F). These Tat^++^ genes were strongly enriched for chromatin regulatory pathways (Fig. 3G). Notably, HIV served as an internal reference point, highlighting the exceptional magnitude of Tat recruitment at the provirus relative to host genes. A corresponding Tat^++^ lncRNA subset (*n* = 54; Supplementary Table S2 ) was also identified (Supplementary Fig. S4C), although functional interpretation was limited by sparse annotation.

Together, these results reveal that Tat associates broadly with host chromatin yet exhibits pronounced quantitative selectivity, with maximal recruitment at the HIV provirus and a restricted subset of host genes.

### Tat-bound host genes exhibit elevated RNAPII and CDK9 occupancy independently of Tat, with Tat-dependent amplification upon stimulation

To determine whether Tat preferentially associates with transcriptionally active chromatin, we generated length- and GC-content-matched control gene sets lacking detectable Tat (Tat^−^) (Supplementary Fig. S5A–C). Genes were then stratified into three classes: Tat^−^ (no Tat peaks), Tat^+^ (Tat-bound genes), and Tat⁺⁺ (Tat-bound genes with ≥2-fold higher Tat ChIP-seq signal in WT + Stim relative to TatNull + Stim).

**Figure 4.**
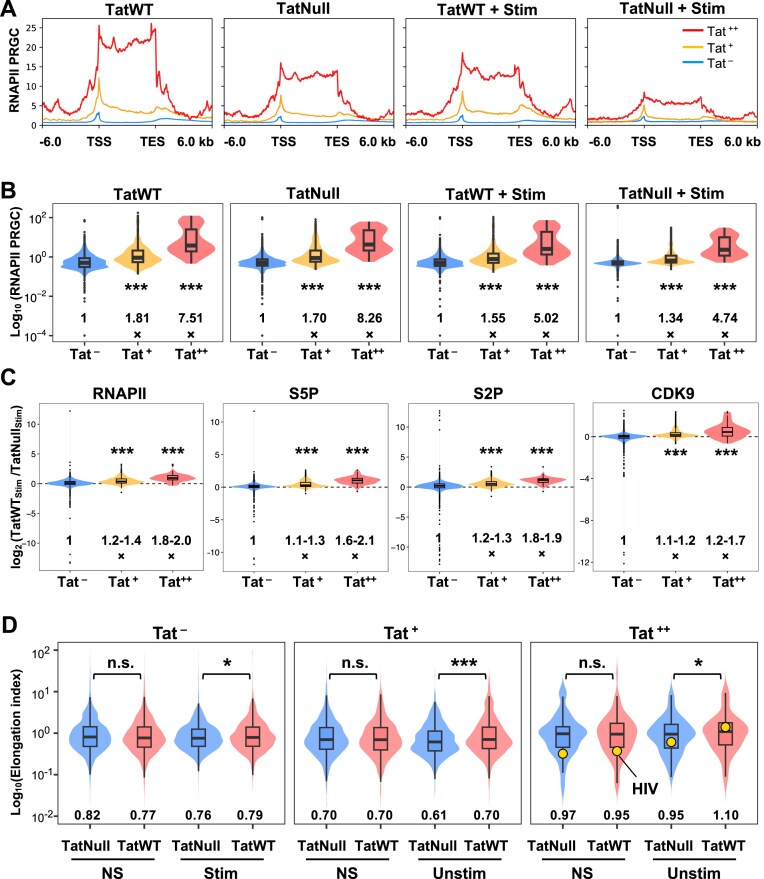
Tat-associated host genes exhibit elevated RNAPII and CDK9 loading independent of RNA output. (**A**) Metagene profiles of RNAPII ChIP-seq signal across PCGs stratified by Tat occupancy (Tat^–^, Tat^+^, Tat^++^), shown for TatWT and TatNull cells under NS and stimulated (PMA + TSA) conditions. Profiles are averaged across scaled gene bodies with ±6 kb flanking regions. (**B**) Gene-level RNAPII occupancy across Tat classes in TatWT and TatNull cells. Violin plots show log_10_ RPGC-normalized, RPGC-normalized RNAPII signal over gene bodies. Boxes denote the interquartile range with the median indicated. Numbers below indicate median fold enrichment relative to Tat^−^ within the same condition. (**C**) Stimulation-induced changes in chromatin occupancy by Tat class. Per-gene Δ-signal values are shown as violin plots for RNAPII, S5P, S2P, and CDK9 across Tat classes. Values below each group indicate the interquartile range (25th–75th percentile) fold change of TatWT/TatNull, normalized to Tat^−^ (linear scale). (**D**) EI across PCGs stratified by Tat occupancy (Tat^−^, Tat^+^, Tat^++^) in TatWT and TatNull cells under NS and Stim conditions. EI was calculated as the ratio of RNAPII S2P signal over the gene body to promoter signal (S2P_body / S2P_promoter) using RPGC-normalized ChIP-seq data and is shown on a log_10_ scale. Violin plots show gene-level EI distributions with median and interquartile range indicated, and the bottom values indicate the median EI of the group. The HIV provirus EI is overlaid as a single point within the Tat^++^ group, calculated using the same metric. Statistical significance was assessed using Wilcoxon rank-sum tests (n.s. no significance, *P < 1 × 10^−3^, ***P < 1 × 10^−10^).

Metagene profiling revealed a stepwise increase in RNAPII occupancy from Tat^−^ to Tat⁺ to Tat⁺⁺ PCGs under unstimulated conditions in both TatWT and TatNull cells (Fig. 4A). Because TatNull cells lack Tat, this hierarchy reflects intrinsic transcriptional engagement at Tat-associated loci rather than a direct Tat effect. CDK9, S2P, and S5P exhibited a similar Tat^−^ < Tat⁺ < Tat⁺⁺ ordering across genotypes (Supplementary Fig. S6A–C), indicating that Tat preferentially associates with chromatin environments already characterized by active transcriptional machinery. Gene-level quantification corroborated these trends: median RNAPII signal increased progressively across Tat classes in all conditions and in both genotypes (Fig. 4B). Stimulation with PMA + TSA globally redistributed RNAPII and reduced the relative separation between classes, yet Tat⁺ and Tat⁺⁺ genes remained consistently more RNAPII-rich than Tat^−^ controls, and this stratification persisted in TatNull samples, reinforcing that they are not established by Tat. These patterns were mirrored in metagene profiles of CDK9, S2P, and S5P (Supplementary Fig. S7).

To isolate Tat-dependent effects while controlling for global stimulation-induced transcription redistribution, we computed per-gene Δ-signals as log_2_ (TatWT + Stim / TatNull + Stim), using TatNull + Stim as matched Tat-deficient reference. Tat⁺ and Tat⁺⁺ PCGs exhibited significantly higher RNAPII, S2P, S5P, and CDK9 Δ-signals than Tat^−^ genes (Fig. 4C), with RNAPII increases of ∼1.3-fold and ∼1.85-fold, respectively. Thus, Tat does not create transcriptional competence *de novo* but instead amplifies recruitment and activity of elongation-associated transcriptional machinery at pre-engaged loci.

We next asked whether these increases translated into greater elongation efficiency by calculating an EI (gene-body/promoter S2P) (Fig. 4D). Under unstimulated conditions, EI values were indistinguishable between TatWT and TatNull cells across all Tat binding classes, indicating that basal elongation efficiency at host genes is largely Tat independent. Following stimulation and Tat accumulation, TatWT cells showed modest EI increases at Tat-associated genes relative to TatNull, most pronounced at Tat⁺ loci. Tat⁺⁺ genes exhibited a similar trend with reduced statistical significance, likely due to already elevated basal EI limiting dynamic range. In contrast, Tat^−^ genes displayed only minor genotype-dependent differences, reflecting with global stimulation effects rather than direct Tat regulation. This modest EI response stands in sharp contrast to the HIV provirus, where EI rose dramatically from comparable WT and TatNull values under basal conditions (0.37 versus 0.32) to a markedly higher EI in TatWT upon stimulation (1.4) (Fig. 2D). Thus, although Tat-dependent increases in S2P occupancy are evident at Tat-bound loci (Fig. 4C), the absence of a focused Tat-TAR recruitment mechanism at host genes constraints how efficiently these increases in RNAPII and P-TEFb occupancy are converted to productive elongation.

Tat-bound lncRNAs displayed analogous behavior. Tat⁺ and Tat⁺⁺ lncRNAs showed elevated RNAPII occupancy in both TatWT and TatNul cells under basal conditions (Supplementary Fig. S8A and B), consistent with a permissive chromatin state. Upon Tat accumulation by stimulation, Tat⁺ and Tat⁺⁺ lncRNAs exhibited significantly increased RNAPII, S2P, S5P, and CDK9 Δ-signals relative to Tat^−^ controls (Supplementary Fig. S8C), yet EI changes were minimal (Supplementary Fig. S8D), suggesting distinct elongation constraints at lncRNAs loci.

Together, these results support a model in which Tat preferentially associates with transcriptionally active chromatin landscapes and selectively amplifies RNAPII and P-TEFb recruitment. Baseline host-driven transcription establishes a permissive state onto which Tat imposes a secondary amplification layer, mirroring the architecture at the HIV provirus, but with far greater elongation control at the virus locus.

### Tat selectively enhances expression at Tat-associated host genes, but with modest magnitude compared to HIV

To determine whether Tat chromatin occupancy translates into functional regulation of host transcription, we analyzed RNA-seq profiles from TatWT and TatNull-infected cells under NS and stimulated conditions (PMA + TSA). Genes were stratified by Tat ChIP-seq binding (Tat^−^, Tat^+^, Tat^++^; Fig. 4). Restricting the analysis to genes with detectable expression yielded 2442 Tat^−^, 823 Tat^+^, and 78 Tat^++^ PCGs, representing the subset of Tat-bound loci that are transcriptionally active.

**Figure 5.**
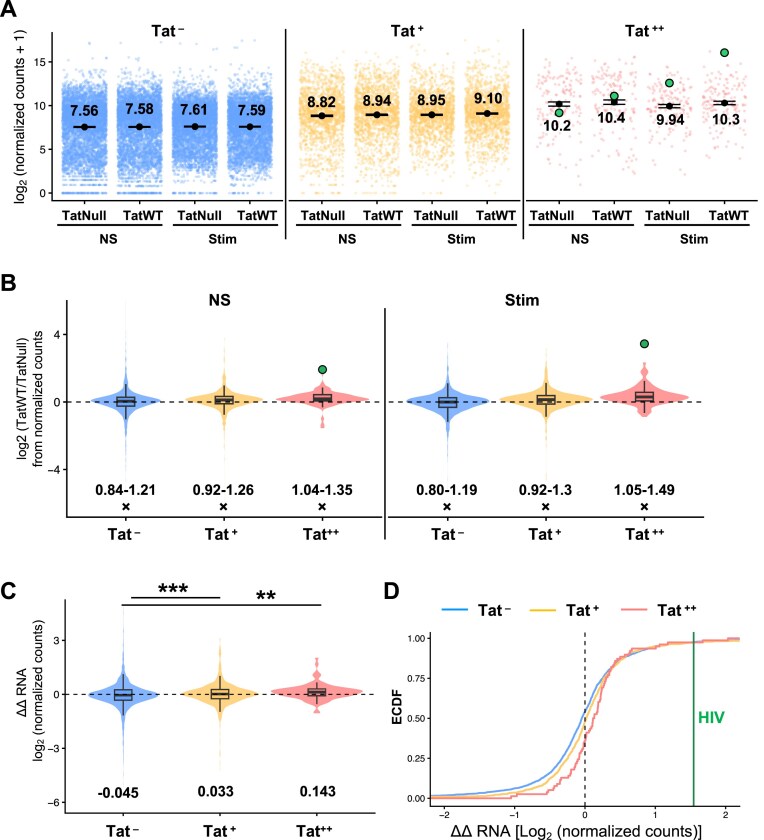
Tat selectively enhances stimulation-dependent transcription at Tat-associated host genes. (**A**) Gene-level expression of host PCGs stratified by Tat chromatin occupancy. Genes were classified as Tat-negative (Tat^−^, *n* = 3461), Tat-bound (Tat^+^, *n* = 894), or high-confidence Tat-associated (Tat^++^, *n* = 65) based on Tat ChIP-seq signal. Expression is shown as log_2_(normalized counts + 1) in TatWT and TatNull cells under NS and stimulated (PMA + TSA; Stim) conditions. Each dot represents an individual gene-sample measurement, whereas *n* denotes the number of unique genes in each Tat group. Black points and horizontal bars indicate the mean ± SEM within each group, with mean values shown above. The HIV provirus is shown as a single green dot for reference and was excluded from host gene quantification and statistical analyses. (**B**) Gene-level Tat dependence of basal and stimulated conditions. Violin plots depict per-gene log_2_ (TatWT/TatNull) values for PCGs grouped by Tat occupancy (Tat ^−^, Tat ^+^, Tat ^++^) under NS and Stim conditions. Overlaid boxplots mark the median and interquartile range; the dashed line indicates no Tat dependence (log_2_ = 0). Values below each group indicate the interquartile range (25th–75th percentile) fold change of TatWT/TatNull, normalized to Tat^−^ (linear scale). (**C**) Tat-dependent stimulation gain (ΔΔRNA) at the gene level. Violin plots show the distribution of ΔΔRNA values stratified by Tat occupancy. Boxplots indicate median and interquartile range; the dashed line denotes ΔΔ = 0 (no Tat-dependent stimulation gain). Statistical comparisons between Tat^−^ and Tat^+^ or Tat^++^ genes were performed using two-sided Wilcoxon rank-sum tests with Benjamini–Hochberg correction (**P < .01, ***P < .001). (**D**) Population-level distribution of Tat-dependent stimulation gain. Empirical cumulative distribution functions of ΔΔ values are shown for PCGs grouped by Tat occupancy. The vertical dashed line indicates ΔΔ = 0. The solid green line marks the ΔΔ value of HIV for scale reference only and was not included in host-gene distributions or statistical analyses. Analysis in panels (B–D) were performed on the same Tat-stratified PCGs set defined in panel (A).

Across all conditions, Tat-associated PCGs (Tat^+^ and Tat^++^) displayed higher absolute expression levels than Tat^−^ genes, including in NS and TatNull infected cells (Fig. 5A). This indicates that Tat binding preferentially occurs at loci already embedded in transcriptionally permissive chromatin rather than establishing basal transcription. Consistent with this interpretation, differences in gene expression between TatWT and TatNull under NS conditions were minimal across all gene classes (median TatWT/TatNull fold change: Tat^−^ = 1.03×, Tat^+^ = 1.08×, Tat^++^ = 1.13×; Fig. 5B), demonstrating that Tat is largely dispensable for basal host gene expression.

In contrast, basal HIV transcription showed a marked Tat dependence. On the same scale used for host genes, the TatWT–TatNull separation at the provirus was 1.92, corresponding to an ~3.8-fold difference (Fig. 5B), highlighting a fundamental distinction between viral and host transcriptional control (Fig. 5B).

Following Tat accumulation upon stimulation, Tat-associated genes exhibited a modest but progressive shift toward higher expression in TatWT relative to TatNull cells, proportional to Tat occupancy (median TatWT/TatNull fold change: Tat^−^ = 0.99×, Tat^+^ = 1.10×, Tat^++^ = 1.23×; Fig. 5B). In contrast, HIV transcription showed a dramatically larger TatWT–TatNull separation of 3.45 (∼11-fold difference), underscoring the disproportionate dependence of viral transcription on Tat.

To quantify Tat-dependent amplification during stimulation, we defined a Tat-dependent stimulation gain metric: ΔΔRNA = (TatWT_Stim_ − TatWT_NS_) − (TatNull_Stim_ − TatNull_NS_). Tat^−^ genes exhibited no Tat-dependent gain (median − 0.045), whereas Tat^+^ and Tat^++^ genes displayed small but significant increases (medians 0.033 and 0.143; Fig. 5C), with cumulative distributions shifting progressively rightward from Tat^−^ to Tat^+^ to Tat^++^ (Fig. 5D). By comparison, HIV exhibited a ΔΔRNA of 1.54, over an order of magnitude larger than host genes. Because HIV already displays substantial Tat dependence at baseline, this metric underestimates Tat’s full effect at the provirus; HIV is shown as a scale reference rather than a direct comparator (Fig. 5D).

lncRNAs showed a qualitatively similar pattern. Tat-associated lncRNAs had higher basal expression than Tat^−^ loci (Supplementary Fig. S9A), with minimal TatWT–TatNull differences under NS conditions (Supplementary Fig. S9B). Following Tat accumulation by stimulation, Tat^+^ and Tat^++^ lncRNAs showed modest WT-biased shifts and limited Tat-dependent gains (Supplementary Fig. S9C and D), with greater heterogeneity than PCGs but similarly small effect sizes.

Collectively, these results demonstrate that Tat preferentially associates with host genes embedded in transcriptionally permissive chromatin and confers only modest amplification of their expression. In contrast, Tat exerts a quantitatively dominant effect at the HIV promoter, revealing a fundamental asymmetry between viral and host transcriptional regulation: Tat acts primarily as a specialized viral amplifier superimposed on a broadly permissive host transcriptional landscape.

### Integrated ChIP-seq and RNA-seq reveal coordinated Tat-associated elongation with limited RNA amplification at host genes

To link Tat-associated chromatin features with transcriptional output, we integrated ChIP-seq and RNA-seq using a ΔΔ framework that isolates the Tat-specific component of the transcriptional response under matched stimulation conditions. RNA-seq analysis was restricted to expressed Tat-bound loci, yielding 978 Tat-bound loci (834 PCGs and 100 lncRNAs). For each gene, ΔΔRNA quantified the additional RNA gain in TatWT relative to TatNull cells after controlling for basal transcription and Tat-independent activation. HIV was included as a reference point for scale.

**Figure 6.**
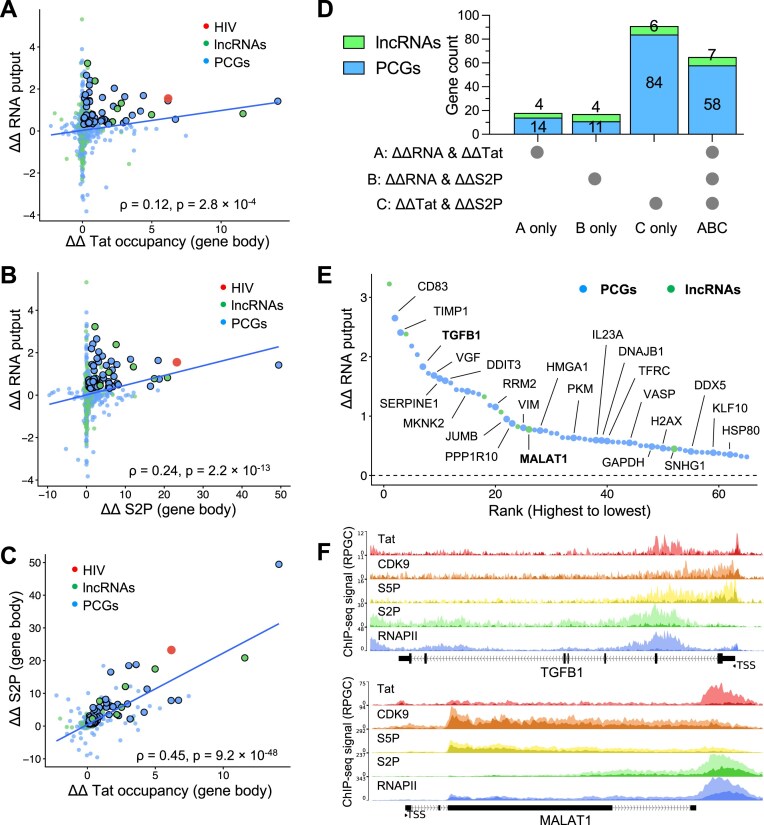
Integrated chromatin and transcriptional analyses reveal coordinated Tat-associated elongation and RNA output at a subset of host genes. (**A**–**C**) Relationships between stimulation-dependent Tat occupancy, elongation-associated RNAPII modification, and RNA output across Tat-bound host genes (*n* = 932 total genes; 832 PCGs and 100 lncRNAs). Scatter plots show ΔΔTat gene-body occupancy versus ΔΔRNA (A), ΔΔS2P gene-body occupancy versus ΔΔRNA (B), and ΔΔTat versus ΔΔS2P (C). Each point represents a Tat-bound PCGs or lncRNAs; the HIV provirus is shown in as a reference point. Linear regression lines are shown for visualization. Spearman correlations were computed using host genes only (HIV excluded). Genes belonging to the intersection set defined in panel (D) are highlighted as enlarged point in panels (A–C). (**D**) Intersection of Tat-regulated host genes exhibiting coordinated increases in Tat occupancy, elongation-associated chromatin features, and RNA output. Genes were ranked by ΔΔRNA, ΔΔTat, and ΔΔS2P, and the top quartiles were intersected. The UpSet plot shows set membership (dots) and gene counts per category (bars), stratified by gene biotype (PCGs and lncRNAs). Genes shared across all three features (A + B + C) define a subset with coordinated Tat-associated elongation and transcriptional gain. (**E**) Ranked distribution of host genes within the A + B + C intersection set. Genes are ordered by ΔΔRNA (highest to lowest), with PCGs and lncRNAs indicated as in panels (A–C). Selected genes previously implicated in HIV-related pathways are highlighted. The dashed horizontal line indicates ΔΔRNA = 0. (**F**) Representative genome browser views of TGFB1 and MALAT1 showing RPGC-normalized ChIP-seq signal for Tat, CDK9, RNAPII, S5P, and S2P, illustrating Tat-associated recruitment of elongation machinery at host genes.

Across host genes, increases in Tat occupancy within gene bodies (ΔΔTat_body) showed only a weak association with ΔΔRNA (Spearman ρ = 0.12; Fig. 6A), and promoter-proximal Tat occupancy was not predictive of RNA gain (Supplementary Fig. S10A). Thus, Tat binding alone is insufficient to explain host transcriptional output. By contrast, elongation-associated chromatin features were more informative: Tat-dependent increases in RNAPII-S2P phosphorylation across gene bodies (ΔΔS2P_body) correlated with ΔΔRNA (ρ = 0.24; Fig. 6B) and were strongly coupled to ΔΔTat_body (ρ = 0.45; Fig. 6C). Similar relationships were observed for CDK9 and total RNAPII occupancy (Supplementary Fig. S10B–D). These results place Tat upstream of elongation-associated RNAPII modification, rather than directly upstream of RNA output.

To identify loci with coordinated Tat-dependent regulation, we ranked Tat-bound genes by ΔΔRNA and intersected the top quartiles of ΔΔRNA, ΔΔTat_body, and ΔΔS2P_body. This revealed a distinct subset of 65 genes (A + B + C), predominantly protein-coding, enriched for loci previously reported to change during HIV infection or to influence the viral life cycle (Fig. 6D and E). TGFB1 and MALAT1 illustrate this pattern [47, 48]: both show stimulation-dependent Tat recruitment together with increased CDK9 and RNAPII occupancy across gene bodies, consistent with Tat-associated engagement of elongation machinery at host genes (Fig. 6F).

Together, these findings demonstrate that Tat recruitment at host genes is tightly coupled to Tat-dependent increases in S2P occupancy that only partially predict RNA output, consistent with limited elongation gains observed at these genes. In contrast, HIV transcription operates in a distinct regime where Tat-dependent elongation is directly translated into disproportionately large RNA amplification. Tat therefore functions not as a global regulator of host transcription, but as a specialized viral amplifier operating within an already permissive host transcriptional landscape.

## Discussion

This study establishes a quantitative, chromatin-preserving framework to disentangle basal host-driven HIV transcription from Tat-dependent amplification. Using an isogenic TatNull HIV_GKO_ system integrated with matched ChIP-seq and RNA-seq analyses, we define the physiological magnitude of Tat-independent transcription and its mechanistic relationship to transcriptional elongation. Across primary CD4⁺ T cells and Jurkat T cells, Tat deficiency restricts proviral output to ∼4%–15% of WT levels, accompanied by proportional reductions in RNAPII occupancy and elongation-associated chromatin marks. At the genome scale, Tat preferentially associates with transcriptionally engaged host loci enriched for RNAPII, CDK9, and elongation signatures, yet its functional impact on host transcription is modest compared with its disproportionate effect at the HIV LTR. These findings support a model in which Tat operates primarily as a selective viral amplifier of transcriptional elongation rather than a global regulator of host gene expression.

Tat is dispensable for promoter engagement but is required for efficient transition into productive elongation. Importantly, Tat loss does not result in accumulation of promoter-proximal paused RNAPII; instead, TatNull proviruses exhibit reduced overall RNAPII occupancy together with a disproportionate defect in gene-body Ser2-phosphorylated RNAPII. In TatWT proviruses, initiation and promoter-proximal RNAPII engagement are efficient, whereas stimulation-induced CDK9 recruitment and accumulation of Ser2-phosphorylated RNAPII across the gene body are markedly attenuated in TatNull cells. Consistent with this, integrative analyses of host genes indicate that elongation-associated chromatin features, rather than Tat occupancy per se, are the strongest predictors of stimulation-dependent RNA output. This placement of Tat upstream of elongation capacity aligns with its canonical role in recruiting P-TEFb and the super elongation complex to promote pause release [2, 3, 49–52]. Efficient elongation likely supports production of full-length genomic RNA and coordinated accumulation of multiply spliced transcripts required for viral gene expression.

The requirement for combined PMA + TSA stimulation further highlights the importance of chromatin accessibility in enabling Tat-independent transcription. Activation of a single pathway (e.g., NF-κB via TNF-α) was insufficient, whereas dual stimulation was required, indicating that Tat-independent transcription arises from convergent regulatory inputs. In TatNull proviruses, limited RNAPII occupancy despite evidence of initiation suggests suboptimal promoter accessibility and/or inneficient PIC assembly. Concurrently, reduced CDK9 recruitment and Ser2 phosphorylation reflect constrained P-TEFb activity, potentially due to incomplete release from the 7SK complex [53, 54]. Together, these findings support a model in which cellular signaling and chromatin accessibility set the level of promoter engagement, while Tat is specifically required to convert these initiation events into productive elongation.

Previous Tat-null models based on single proviral integrations demonstrated the indispensability of Tat for sustained HIV transcription, but could not capture the integration-site diversity and chromatin heterogeneity that shape proviral behavior [55–60]. By quantifying transcription across thousands of independently integrated proviruses, including in acutely infected primary CD4⁺ T cells, the TatNull HIV_GKO_ platform defines the magnitude of Tat-independent transcription within a fully chromatinized genomic context. This quantitative resolution is particularly relevant for Tat-targeted therapeutic strategies, which suppress but do not abolish viral transcription and therefore require an accurate understanding of residual Tat-independent activity.

Importantly, interpretation of these measurements is influenced by cellular context. The primary CD4⁺ T-cell system used here reflects an activated or transitioning state rather than a fully quiescent latency model, as bona fide latency in primary cells requires entry into a non-permissive, quiescent memory state with globally reduced transcriptional activity [61]. Accordingly, the basal transcription observed likely reflects ongoing chromatin engagement in an activated environment rather than true latency.

Although Tat is classically described as binding TAR RNA at the HIV promoter, our ChIP-seq analyses reveal Tat occupancy across the provirus and at a defined subset of host loci. At the provirus, Tat-signal is strongly TSS-centric, consistent with focused TAR-dependent recruitment. In contrast, Tat binding at host genes lacks a dominant peak and broadly overlaps regions enriched for RNAPII and elongation marks, indicating association with transcriptionally active chromatin rather sequence-specific targeting. Given Tat’s lack of intrinsic DNA-binding activity and its extensive interactions with RNAPII and P-TEFb [36, 49, 62], Tat is likely recruited to host chromatin through protein–protein interactions. Within this framework, Tat associates with pre-engaged transcriptional complexes at host loci but exerts only modest effects on transcription output. In contrast, Tat recruitment at the HIV provirus is spatially focused and functionally dominant, revealing a fundamental asymmetry between viral and host transcriptional regulation. While Tat has been reported to interact with chromatin remodeling complexes such as PBAF/SWI-SNF [7], our genome-wide data indicate that Tat predominantly co-localizes with RNAPII-P-TEFb elongation complexes at both viral and host loci, with its functional impact dictated by the mode of recruitment: TAR-dependent engagement at the HIV LTR drives efficient and sustained transcriptional elongation, whereas association with RNAPII-P-TEFb at host genes results in more modest and context-dependent transcriptional amplification.

Reassessing Tat’s influence on host transcription under physiological expression levels further clarifies this asymmetry. Endogenous Tat was detectable at a defined subset of host loci, predominantly PCGs, with fewer lncRNAs, consistent with earlier reports of Tat occupancy at active chromatin [45, 46]. However, Tat-associated transcriptional modulation was substantially smaller than previously reported in Tat overexpression systems [45], which likely distorted regulatory stoichiometry. Under physiological conditions, Tat confers only modest stimulation-dependent RNA gains at host loci and limited effects at lncRNAs, indicating that Tat does not broadly reprogram host transcription.

Although downstream phenotypes were not directly assessed, the selective upregulation of Tat-associated genes, particularly those involved in chromatin organization, stress responses, and cellular regulation, suggests potential functional consequences. For example, strongly Tat-responsive genes (e.g. TGFB1, SERPINE1) are linked to signaling, survival, and immune modulation pathways, which may influence cellular fitness and viral output between TatWT and TatNull conditions. However, given the modest and restricted nature of these effects, any phenotypic consequences are likely indirect and arise from cumulative changes in specific genes rather than broad reprogramming of cell identity. Further functional studies will be required to define these effects.

Several considerations inform these conclusions. Cellular stimulation induces global transcriptional remodeling that may compress Tat-dependent differences at host loci when assessed at the population level, and indirect effects of other viral proteins cannot be fully excluded. Nonetheless, by integrating chromatin occupancy with direct measurements of RNA output, our analyses provide a conservative and functionally grounded assessment of Tat-dependent regulation. This approach is particularly important for lncRNAs and other regulatory elements whose transcriptional consequences are difficult to infer from chromatin features alone.

A major strength of this work is the use of the isogenic TatNull HIV_GKO_ platform, which preserves native proviral chromatin architecture while enabling quantitative dissection of Tat-dependent and Tat-independent transcription across heterogeneous integration sites. This system provides a generalizable framework for interrogating early viral transcription and host regulatory pathways that operate independently of Tat. For example, BRD4, a key modulator of HIV transcription, influences proviral output in both TatWT and TatNull contexts, thereby separating Tat-dependent from Tat-independent mechanisms of regulation [35, 63–65]. Such applications illustrate how the TatNull platform can be leveraged to resolve host-virus regulatory interactions and identify host factors that sustain transcription in the absence of Tat.

Together, our findings refine current models of HIV transcription by defining the physiological magnitude of Tat-independent expression and by revealing a fundamental asymmetry between viral and host transcriptional regulation. Tat acts not as a broad host transcriptional regulator, but as a specialized viral amplifier that exploits pre-existing transcriptionally permissive chromatin to drive disproportionately large increases in proviral output. Identifying the host factors and chromatin regulators that sustain this residual transcription will be critical for understanding how HIV persists under conditions of partial transcriptional repression. The TatNull HIV_GKO_ framework offers a versatile platform for probing early transcriptional regulation and informing strategies aimed at achieving durable transcriptional silencing of HIV.

## Supplementary Material

gkag631_Supplemental_File

## Data Availability

Sequencing data have been deposited in the Gene Expression Omnibus (GEO) under accession number GSE318861. Data will be released upon publication.
