# Supplementary material for: Quantitative limits of host-driven HIV transcription and host gene control by the viral transactivator Tat
Source: Nucleic Acids Res. 2026 Jun 22;54(12):gkag631. doi: 10.1093/nar/gkag631 (PMC13284711; doi:10.1093/nar/gkag631)
Supplement: gkag631_Supplemental_File [file gkag631_supplemental_file.pdf]

Figure S1. Construction and validation of Tat mutated HIV_{GKO} constructs and infection controls.

(A) Schematic of Tat-mutated HIV_{GKO} constructs.

(B) Tat-dependent transcriptional activity was quantified using HeLa-LTR-Luc reporter cells transfected with TatWT, TatLeak, or TatNull HIV_{GKO} constructs. Luciferase activity was measured 48 h post-transfection and normalized to mKO2 fluorescence to control for transfection efficiency. Data represent the mean ± s.e.m. of three independent biological replicates. One-way ANOVA, ****p < 0.0001.

(C-D) Detection of viral protein expression by GFP and intracellular p24 staining. (C) Representative flow cytometry plots at 3 days post-infection (dpi) showing GFP or p24 staining in cells infected with TatWT, TatLeak, or TatNull HIVGKO viruses. Cells harboring integrated provirus were defined as GFP^+ or $mKO2^+$, and GFP or p24 signals were analyzed within this population. (D) Relative mean fluorescence intensity (MFI) of GFP and p24, normalized to TatWT.

(E-F) Flow cytometry analysis of infected cell populations over time using GFP as a marker of HIV expression. (E) Representative gating strategy at 3 and 6 days post-infection (dpi), showing GFP versus mKO2 to define infected cells. Cells harboring integrated provirus were identified as GFP^+ or $mKO2^+$. (F) Time course of the percentage of integrated cells from 3–18 dpi across three donors.

(G) Integration controls showing comparable infection efficiency across TatWT, TatLeak, and TatNull HIV_{GKO} viruses in Jurkat $CD4^+$ T cells, based on $mKO2^+$ frequencies measured by flow cytometry on 4dpi.

Figure S2. ChIP-seq sample characterization.

(A) Flow cytometry profiles of TatWT and TatNull HIV_{GKO} populations before and after PMA+TSA stimulation.

(B) Relative HIV mRNA levels normalized to RPL13A corresponding to samples in (A). Data represent the mean ± s.e.m. of three independent biological replicates. The % of RNA level in TatNull to TatWT is labeled.

(C) Western blot verifying Tat expression in TatWT but not TatNull HIV-integrated cells.

(D) Sanger sequencing result of the HIV cDNA amplified from the total RNA of indicated Jurkat population.

(E) Cell cycle analysis by flow cytometry using propidium iodide (PI) staining. Cells were fixed, stained with PI, and analyzed by flow cytometry to measure DNA content. Histograms show DNA content profiles with G0/G1, S, and G2/M phases indicated under non-stimulated (NS) and stimulated (Stim) conditions.

Figure S3. Validation of Tat ChIP-seq reproducibility and transcription-factor co-localization at high-confidence Tat peaks.

(A) Replicate concordance heatmap for Tat ChIP-seq. Pairwise Spearman correlations were calculated from library-size-normalized Tat ChIP-seq signal summarized in 10-kb genomic bins using deepTools multiBamSummary. Heatmaps display correlation coefficients across biological replicates spanning four experimental conditions (two replicates per condition), demonstrating strong within-condition reproducibility.

(B) Tat peak overlap analysis. Tat peaks (n = 1,468; called using TatNull as the background control) were intersected with RNAPII, S2P, S5P, and CDK9 ChIP-seq peak sets using bedtools intersect. Top: percentage of Tat peaks overlapping each factor. Bottom: observed overlaps compared with 100 shuffled genomic backgrounds generated using bedtools shuffle (excluding blacklist regions).

(C) ChIP-seq signal quantification at Tat peaks. RPGC-normalized signal for Tat, RNAPII, S2P, S5P, and CDK9 was extracted at all Tat peaks using deepTools multiBigwigSummary (BED-file mode). Log_{10}-transformed values are shown as scatterplots, with each point representing a Tat peak. Linear regression lines (red) are shown for visualization.

Figure S4. Functional annotation of Tat-bound host genes.

(A,B) GO Biological Process enrichment of Tat-bound protein-coding genes (PCGs; n = 856) and Tat-bound lncRNAs (n = 124). Dot plots display the top enriched GO terms ranked by adjusted p value. GeneRatio indicates the fraction of Tat-bound genes assigned to each term; dot size represents gene count and dot color denotes the Benjamini-Hochberg-adjusted p value.

(C) Scatterplot of Tat ChIP-seq signal (RPGC) in TatWT cells versus TatNull cells for all Tat-bound lncRNAs. Tat^{+} (n=267) and Tat^{++} (n=54) gene classes are indicated, and the top ten Tat^{++} genes are labeled. The HIV provirus is shown as a green reference point.

Figure S5. Tat-bound and non-bound genes show similar length and GC-content distributions.

(A) Gene-length distributions for protein-coding genes (PCGs; Tat^{+} n = 894, Tat^{-} n = 3,461) and lncRNAs (Tat^{+} n = 267, Tat^{-} n = 1,038). Genes were grouped into $\log_{10}$-scaled length bins, and the fraction of genes per bin was plotted for Tat-bound (Tat^{+}) and non-Tat control (Tat^{-}) gene sets.

(B) Gene-length distributions for Tat^{+} and Tat^{-} PCGs and lncRNAs, shown separately.

(C) GC-content distributions for Tat^{+} and Tat^{-} PCGs and lncRNAs. GC content was calculated using bedtools nuc, and distributions were visualized using violin plots (median indicated) and density plots. Protein-coding and lncRNA genes are shown in separate panels.

Figure S6. Supporting analyses for Figure 4: Metagene profiles of CDK9, S2P, and S5P at Tat-stratified PCGs.

Genome-wide averaged ChIP-seq signal was computed for Tat^{-}, Tat^{+}, and Tat^{++} protein-coding gene groups using deepTools computeMatrix (scale-regions mode), with scaled gene bodies and ±6 kb unscaled flanking regions. Profiles span −6 kb relative to TSS, 6 kb scaled gene body, and +6 kb relative to TES. Profiles are shown for TatWT, TatNull, TatWT+Stim, and TatNull+Stim conditions. Each panel displays averaged RPGC-normalized ChIP-seq signal for, CDK9 (A), S2P (B), and S5P (C).

Figure S7. Supporting analyses for Figure 4: RPGC violin plots of CDK9, S2P-RNAPII and S5P-RNAPII across four conditions.

Gene-level RPGC values were quantified from RPGC-normalized bigWig files for Tat^{-}, Tat^{+}, and Tat^{++} gene groups across TatWT, TatNull, TatWT+Stim, and TatNull+Stim conditions. Violin plots show $\log_{10}$-scaled RPGC distributions for CDK9 (A), S2P (B), and S5P (C). Median fold-change

values represent Tat^+ or Tat^{++} relative to Tat^- within each condition. Statistical comparisons were performed using Wilcoxon rank-sum tests (***$p < 1 \times 10^{-10}$).

Figure S8. lncRNAs exhibit Tat-dependent RNAPII and cofactor recruitment similar to protein-coding genes.

(A) Metagene profiles of and RNAPII across lncRNAs classified as Tat^-, Tat^+, or Tat^{++}. Profiles are shown for TatWT and TatNull cells under non-stimulated and PMA+TSA-stimulated conditions.

(B) RNAPII RPGC violin plots. Gene-level RNAPII values were extracted from RPGC-normalized bigWig files over gene bodies for TatWT and TatNull samples. Distributions are shown for Tat^-, Tat^+, and Tat^{++} groups with $\log_{10}$ scaling. Fold changes were calculated using group medians relative to Tat^-.

(C) Δ-signal violin plots for RNAPII, S2P, S5P, and CDK9 across Tat classes. Fold changes reflect median Δ-signal values for Tat^+ or Tat^{++} relative to Tat^-.

(D) Elongation index (EI) across protein-coding genes stratified by Tat occupancy (Tat^-, Tat^+, Tat^{++}) in TatWT and TatNull cells under NS and Stim conditions. EI was calculated as the ratio of RNAPII S2P signal over the gene body to promoter signal (S2P_body / S2P_promoter) using RPGC-normalized ChIP-seq data and is shown on a log10 scale. Violin plots show gene-level EI distributions with median and interquartile range indicated, and the bottom values indicate the median EI of the group.

Statistical significance was assessed using Wilcoxon rank-sum tests (*$p < 1\times10^{-3}$, ** $p< 1\times10^{-5}$, *** $p<1 \times10^{-10}$).

Figure S9. Tat-dependent transcriptional response at Tat-associated host lncRNAs.

(A) Gene-level expression of host lncRNAs stratified by Tat chromatin occupancy. Genes were classified as Tat^- (n=267), Tat^+ (n=162), or Tat^{++} (n=37) based on Tat ChIP-seq signal. Expression is shown as $\log_2$(normalized counts + 1) in TatWT and TatNull cells under non-stimulated (NS) and stimulated (PMA+TSA; Stim) conditions. Each dot represents an individual gene-sample measurement, whereas n denotes the number of unique genes in each Tat group. Black points

and horizontal bars indicate the mean ± s.e.m., with mean values shown above. The HIV provirus is shown as a single green dot for reference and excluded from host-gene statistical analyses.

(B) Gene-level Tat dependence of basal and stimulated conditions. Violin plots depict per-gene differences ($\log_2$ scale) grouped by Tat occupancy. Numbers below each group indicate the interquartile range fold change of (TatWT/TatNull) on the linear scale.

(C) Tat-dependent stimulation gain (ΔΔRNA) at the gene level. Violin plots show ΔΔRNA distributions stratified by Tat occupancy; dashed line indicates ΔΔRNA = 0. Statistical comparisons were performed using two-sided Wilcoxon rank-sum tests with Benjamini–Hochberg correction (*$p < 0.05$, ***$p < 0.001$).

(D) Population-level ECDFs of ΔΔRNA values for lncRNAs grouped by Tat occupancy. The vertical dashed line denotes ΔΔRNA = 0; the HIV provirus is shown for scale reference only.

Analysis in B-D were performed on the same Tat-stratified PCGs set defined in A.

Figure S10. Relationships between stimulation-dependent Tat occupancy, RNAPII Ser2 phosphorylation, and RNA output across Tat-bound host genes.

Scatter plots show relationships between ΔΔTat occupancy at promoters versus ΔΔRNA (A), ΔΔCDK9 gene-body occupancy versus ΔΔRNA (B), ΔΔRNAPII gene-body occupancy versus ΔΔRNA (C), and ΔΔS2P gene-body occupancy versus ΔΔRNA (D), using the same set of Tat-bound host genes analyzed in Figure 6A-C. Each point represents a Tat-bound protein-coding gene (PC, blue) or lncRNA (green); the HIV provirus is shown in red for reference. Linear regression lines are shown for visualization. Spearman correlations were computed using host genes only (HIV excluded). Genes belonging to the intersection set defined in Figure 6D are highlighted as enlarged points.

Supplementary Tables:

Table S1. The Tat ChIP signal of Tat++ protein coding genes (PCGs)

Table S2. The Tat ChIP signal of Tat++ lncRNAs.

Figure S1

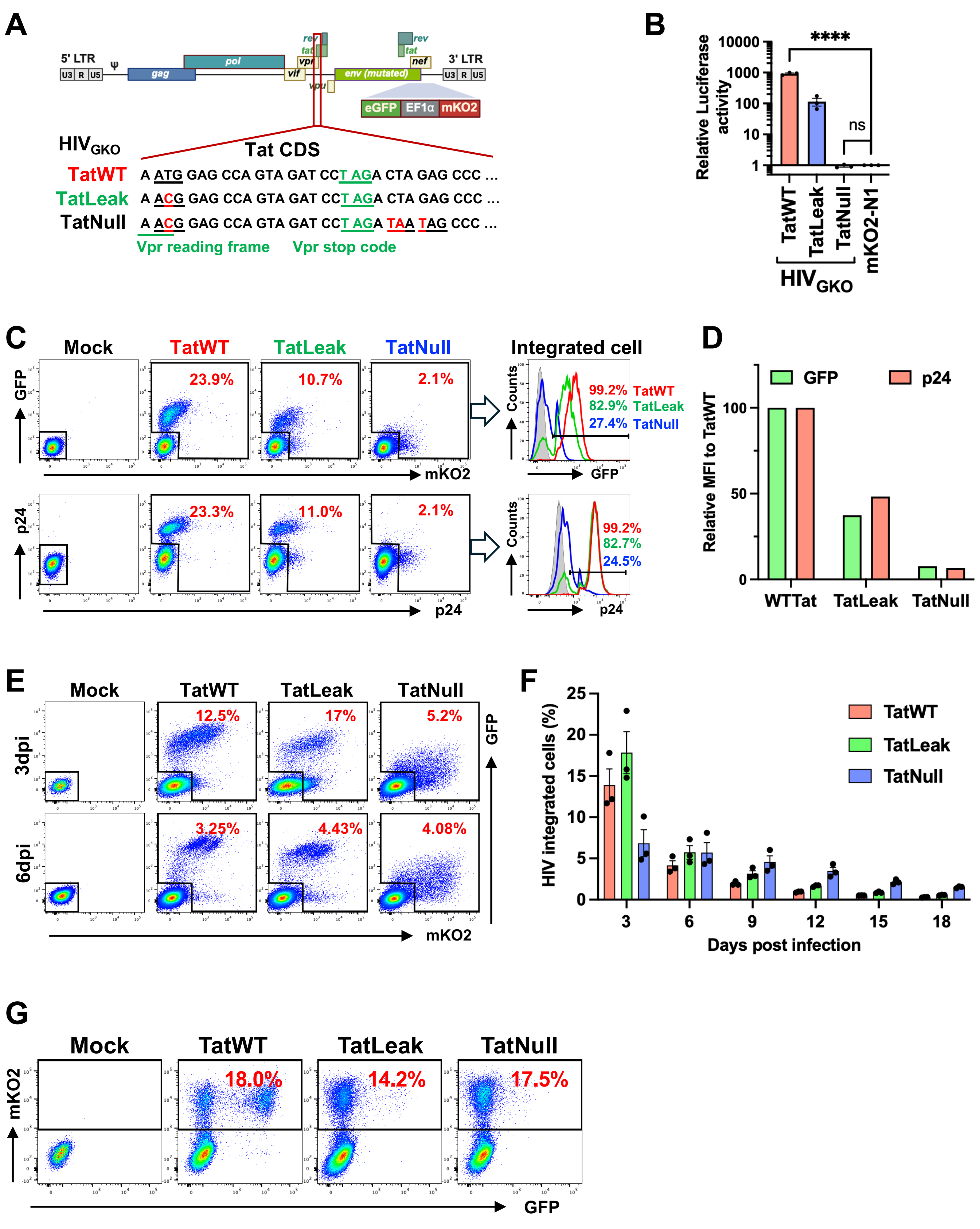

A
5' LTR
3' LTR
gag
pol
vif
vpr
tat
rev
vpu
env (mutated)
nef
eGFP
EF1α
mKO2
HIVGKO
Tat CDS
TatWT A ATG GAG CCA GTA GAT CCT AGA CTA GAG CCC ...
TatLeak A ACG GAG CCA GTA GAT CCT AGA CTA GAG CCC ...
TatNull A ACG GAG CCA GTA GAT CCT AGA TAA TAG CCC ...
Vpr reading frame
Vpr stop code
B
Relative Luciferase activity
****
ns
TatWT
TatLeak
TatNull
mKO2-N1
HIVGKO
C
Mock
TatWT
TatLeak
TatNull
Integrated cell
GFP
mKO2
p24
Counts
23.9%
10.7%
2.1%
99.2% TatWT
82.9% TatLeak
27.4% TatNull
23.3%
11.0%
2.1%
99.2%
82.7%
24.5%
D
GFP
p24
Relative MFI to TatWT
WTTat
TatLeak
TatNull
E
Mock
TatWT
TatLeak
TatNull
3dpi
6dpi
12.5%
17%
5.2%
3.25%
4.43%
4.08%
GFP
mKO2
F
HIV integrated cells (%)
Days post infection
TatWT
TatLeak
TatNull
G
Mock
TatWT
TatLeak
TatNull
mKO2
GFP
18.0%
14.2%
17.5%

Figure S2

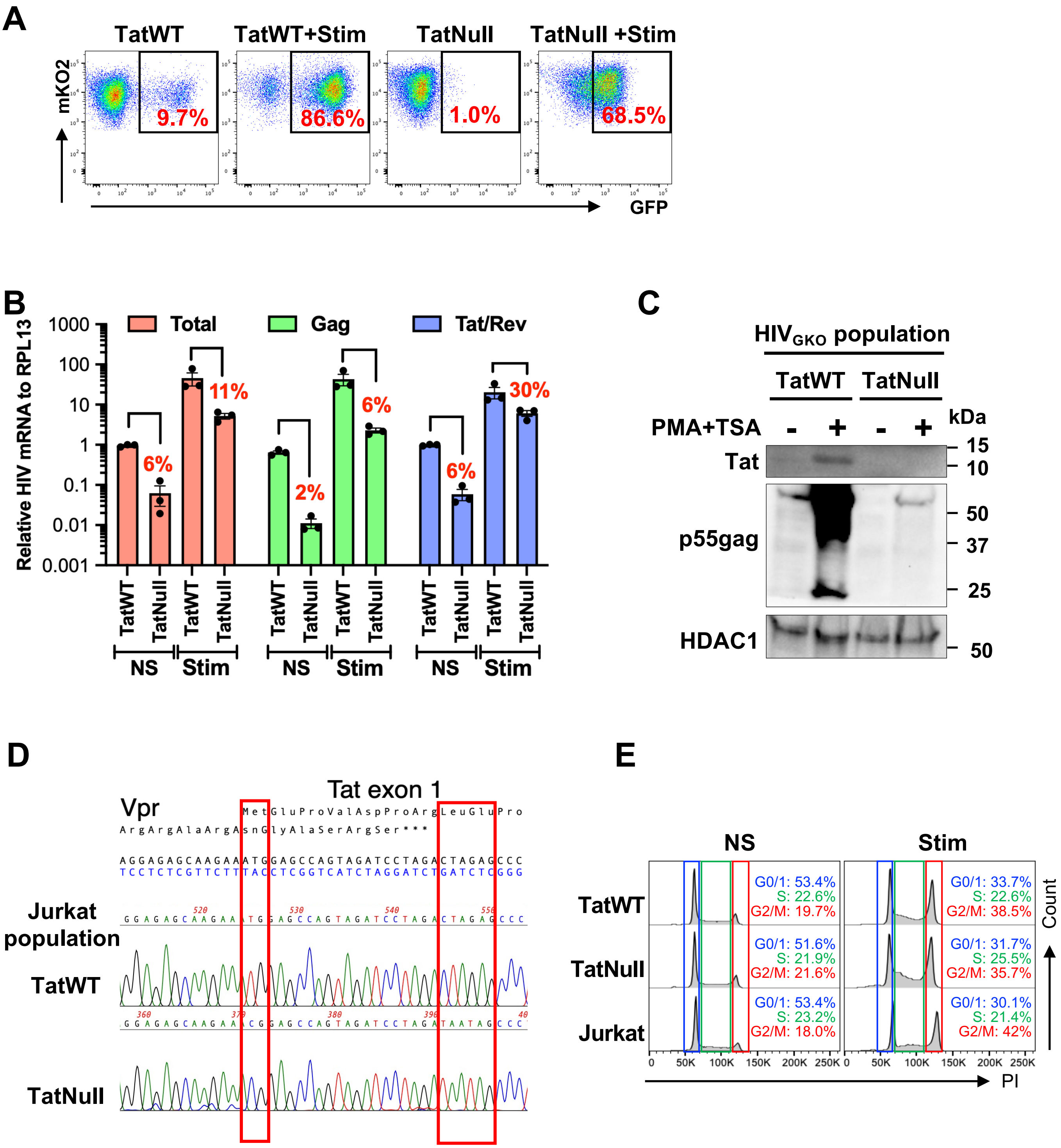
A
TatWT
TatWT+Stim
TatNull
TatNull +Stim
mKO2
GFP
9.7%
86.6%
1.0%
68.5%
B
Total
Gag
Tat/Rev
Relative HIV mRNA to RPL13
1000
100
10
1
0.1
0.01
0.001
6%
11%
2%
6%
6%
30%
TatWT
TatNull
NS
Stim
C
HIVGKO population
TatWT
TatNull
PMA+TSA
-
+
kDa
Tat
p55gag
HDAC1
15
10
50
37
25
50
D
Vpr
Tat exon 1
MetGluProValAspProArgLeuGluPro
ArgArgAlaArgAsnGlyAlaSerArgSer***
AGGAGAGCAAGAAATGGAGCCAGTAGATCCTAGACTAGAGCCC
TCCTCTCGTTCTTTACCTCGGTCATCTAGGATCTGATCTCGGG
Jurkat population
TatWT
TatNull
E
NS
Stim
TatWT
TatNull
Jurkat
G0/1: 53.4%
S: 22.6%
G2/M: 19.7%
G0/1: 33.7%
S: 22.6%
G2/M: 38.5%
G0/1: 51.6%
S: 21.9%
G2/M: 21.6%
G0/1: 31.7%
S: 25.5%
G2/M: 35.7%
G0/1: 53.4%
S: 23.2%
G2/M: 18.0%
G0/1: 30.1%
S: 21.4%
G2/M: 42%
Count
PI
0
50K
100K
150K
200K
250K

Figure S3

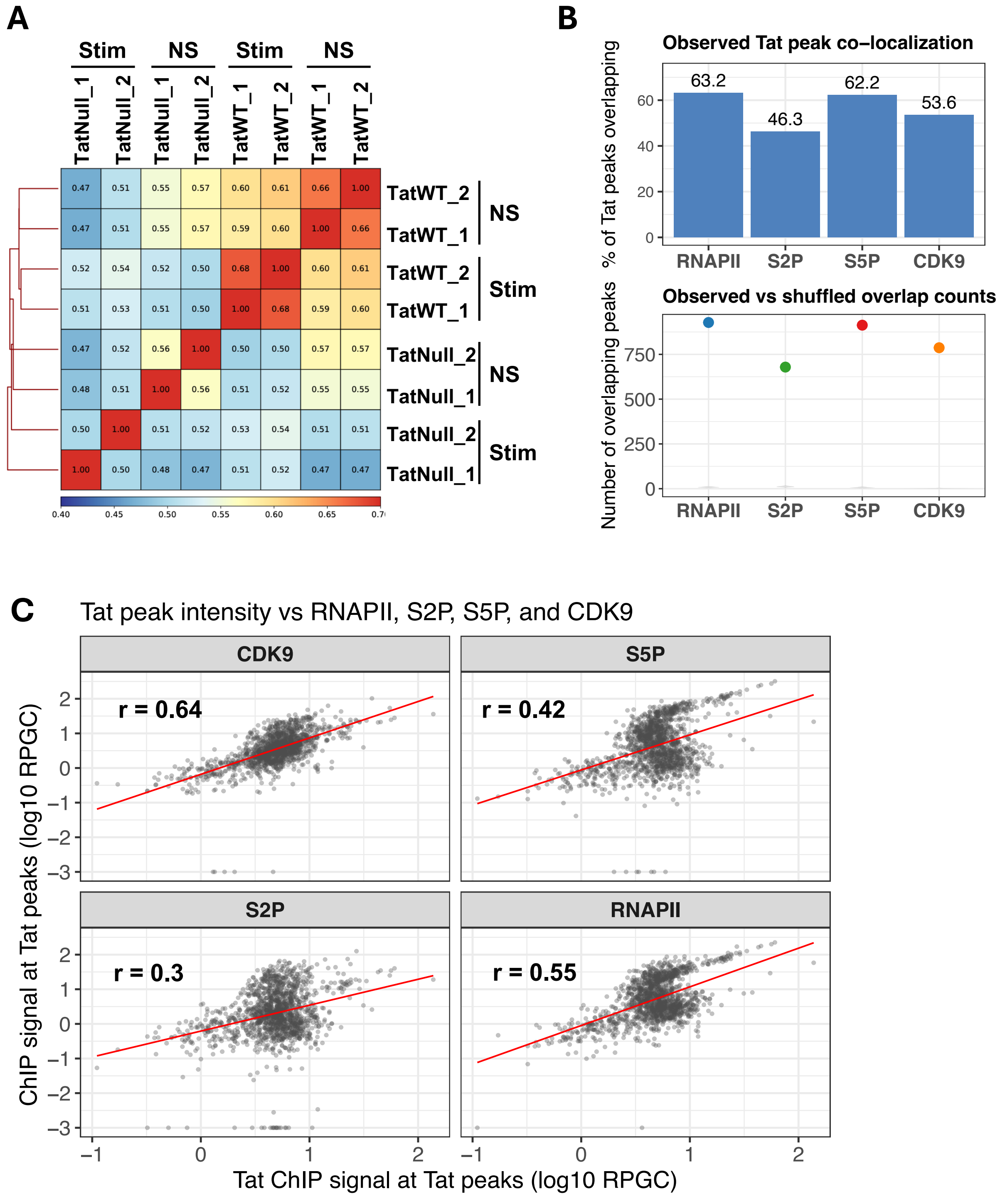
A
Stim
NS
Stim
NS
TatNull_1
TatNull_2
TatNull_1
TatNull_2
TatWT_1
TatWT_2
TatWT_1
TatWT_2
0.47 0.51 0.55 0.57 0.60 0.61 0.66 1.00
0.47 0.51 0.55 0.57 0.59 0.60 1.00 0.66
0.52 0.54 0.52 0.50 0.68 1.00 0.60 0.61
0.51 0.53 0.51 0.50 1.00 0.68 0.59 0.60
0.47 0.52 0.56 1.00 0.50 0.50 0.57 0.57
0.48 0.51 1.00 0.56 0.51 0.52 0.55 0.55
0.50 1.00 0.51 0.52 0.53 0.54 0.51 0.51
1.00 0.50 0.48 0.47 0.51 0.52 0.47 0.47
TatWT_2
TatWT_1
NS
TatWT_2
TatWT_1
Stim
TatNull_2
TatNull_1
NS
TatNull_2
TatNull_1
Stim
0.40 0.45 0.50 0.55 0.60 0.65
B
Observed Tat peak co−localization
% of Tat peaks overlapping
63.2
46.3
62.2
53.6
0 20 40 60
RNAPII S2P S5P CDK9
Observed vs shuffled overlap counts
Number of overlapping peaks
0 250 500 750
RNAPII S2P S5P CDK9
C
Tat peak intensity vs RNAPII, S2P, S5P, and CDK9
CDK9
r = 0.64
S5P
r = 0.42
S2P
r = 0.3
RNAPII
r = 0.55
ChIP signal at Tat peaks (log10 RPGC)
2 1 0 −1 −2 −3
−1 0 1 2
Tat ChIP signal at Tat peaks (log10 RPGC)

Figure S4

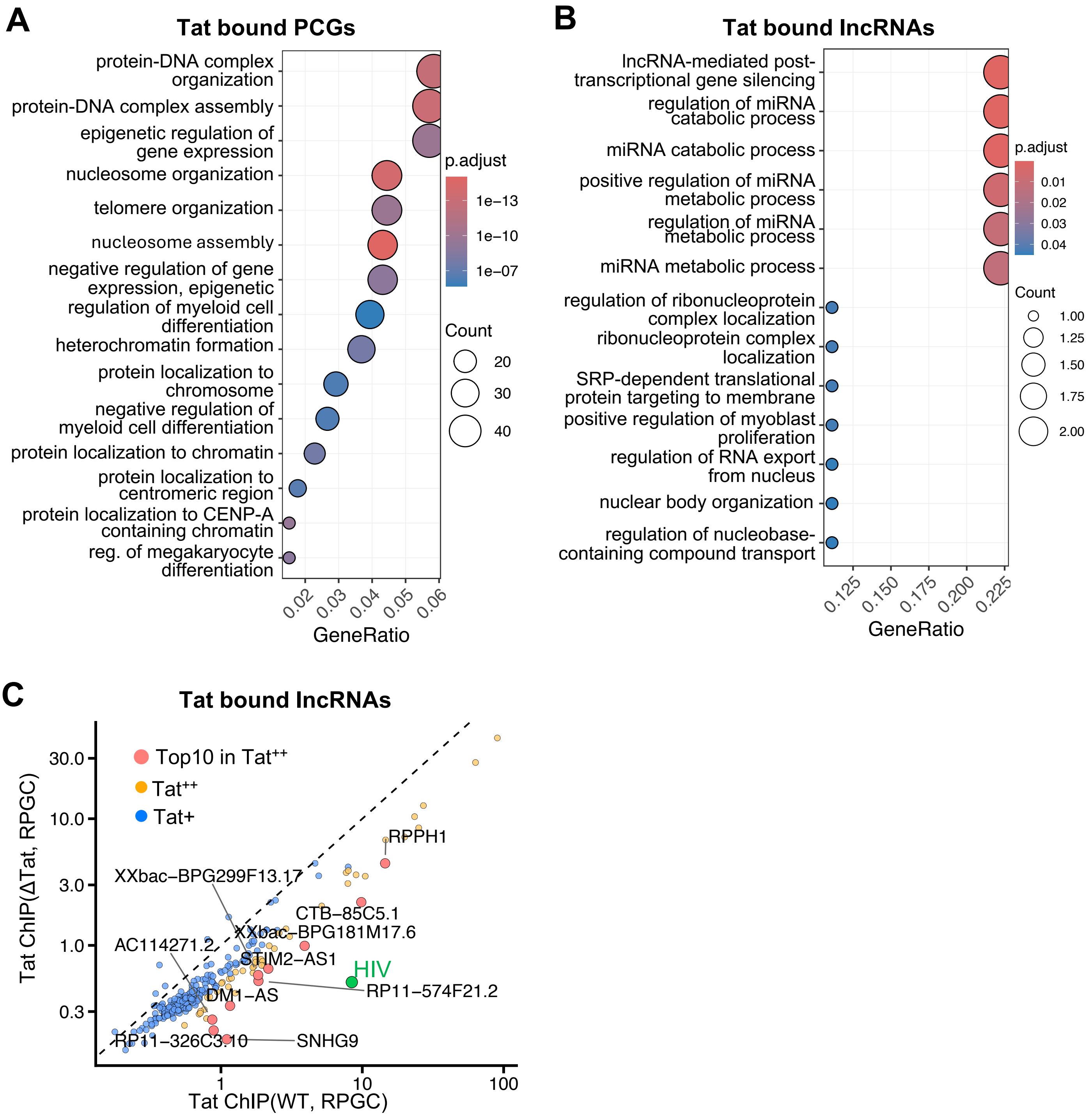
A
Tat bound PCGs
protein-DNA complex organization
protein-DNA complex assembly
epigenetic regulation of gene expression
nucleosome organization
telomere organization
nucleosome assembly
negative regulation of gene expression, epigenetic
regulation of myeloid cell differentiation
heterochromatin formation
protein localization to chromosome
negative regulation of myeloid cell differentiation
protein localization to chromatin
protein localization to centromeric region
protein localization to CENP-A containing chromatin
reg. of megakaryocyte differentiation
0.02 0.03 0.04 0.05 0.06
GeneRatio
p.adjust
1e−13
1e−10
1e−07
Count
20
30
40
B
Tat bound lncRNAs
lncRNA-mediated post-transcriptional gene silencing
regulation of miRNA catabolic process
miRNA catabolic process
positive regulation of miRNA metabolic process
regulation of miRNA metabolic process
miRNA metabolic process
regulation of ribonucleoprotein complex localization
ribonucleoprotein complex localization
SRP-dependent translational protein targeting to membrane
positive regulation of myoblast proliferation
regulation of RNA export from nucleus
nuclear body organization
regulation of nucleobase-containing compound transport
0.125 0.150 0.175 0.200 0.225
GeneRatio
p.adjust
0.01
0.02
0.03
0.04
Count
1.00
1.25
1.50
1.75
2.00
C
Tat bound lncRNAs
Top10 in Tat++
Tat++
Tat+
RPPH1
XXbac−BPG299F13.17
CTB−85C5.1
XXbac−BPG181M17.6
AC114271.2
STIM2−AS1
HIV
RP11−574F21.2
DM1−AS
RP11−326C3.10
SNHG9
30.0
10.0
3.0
1.0
0.3
Tat ChIP(ΔTat, RPGC)
1
10
100
Tat ChIP(WT, RPGC)

Figure S5

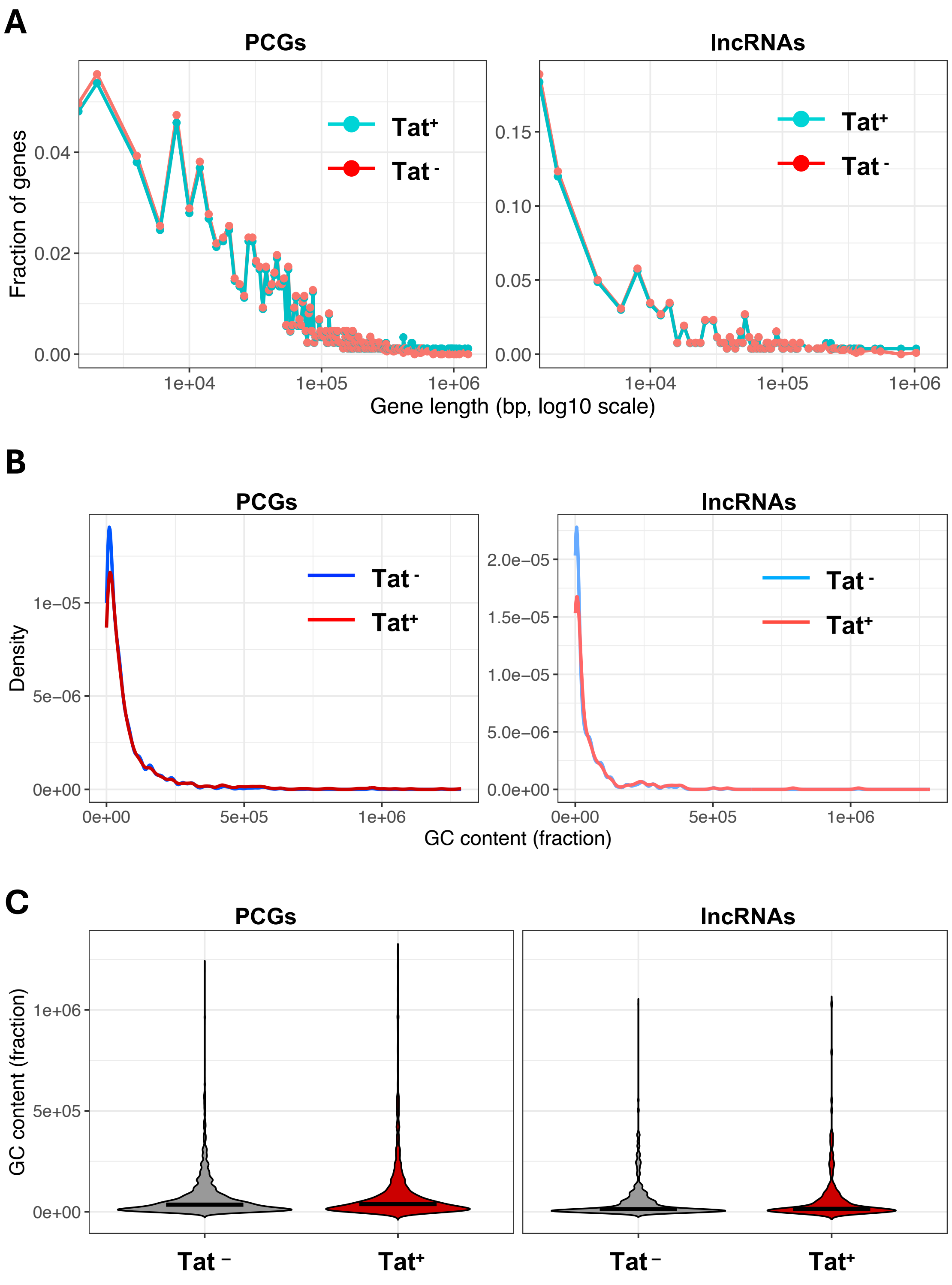

A
PCGs
lncRNAs
Tat+
Tat -
Fraction of genes
0.00
0.02
0.04
0.05
0.10
0.15
1e+04
1e+05
1e+06
Gene length (bp, log10 scale)
B
PCGs
lncRNAs
Tat -
Tat+
Density
0e+00
5e−06
1e−05
0.0e+00
5.0e−06
1.0e−05
1.5e−05
2.0e−05
0e+00
5e+05
1e+06
GC content (fraction)
C
PCGs
lncRNAs
GC content (fraction)
0e+00
5e+05
1e+06
Tat −
Tat+

Figure S6

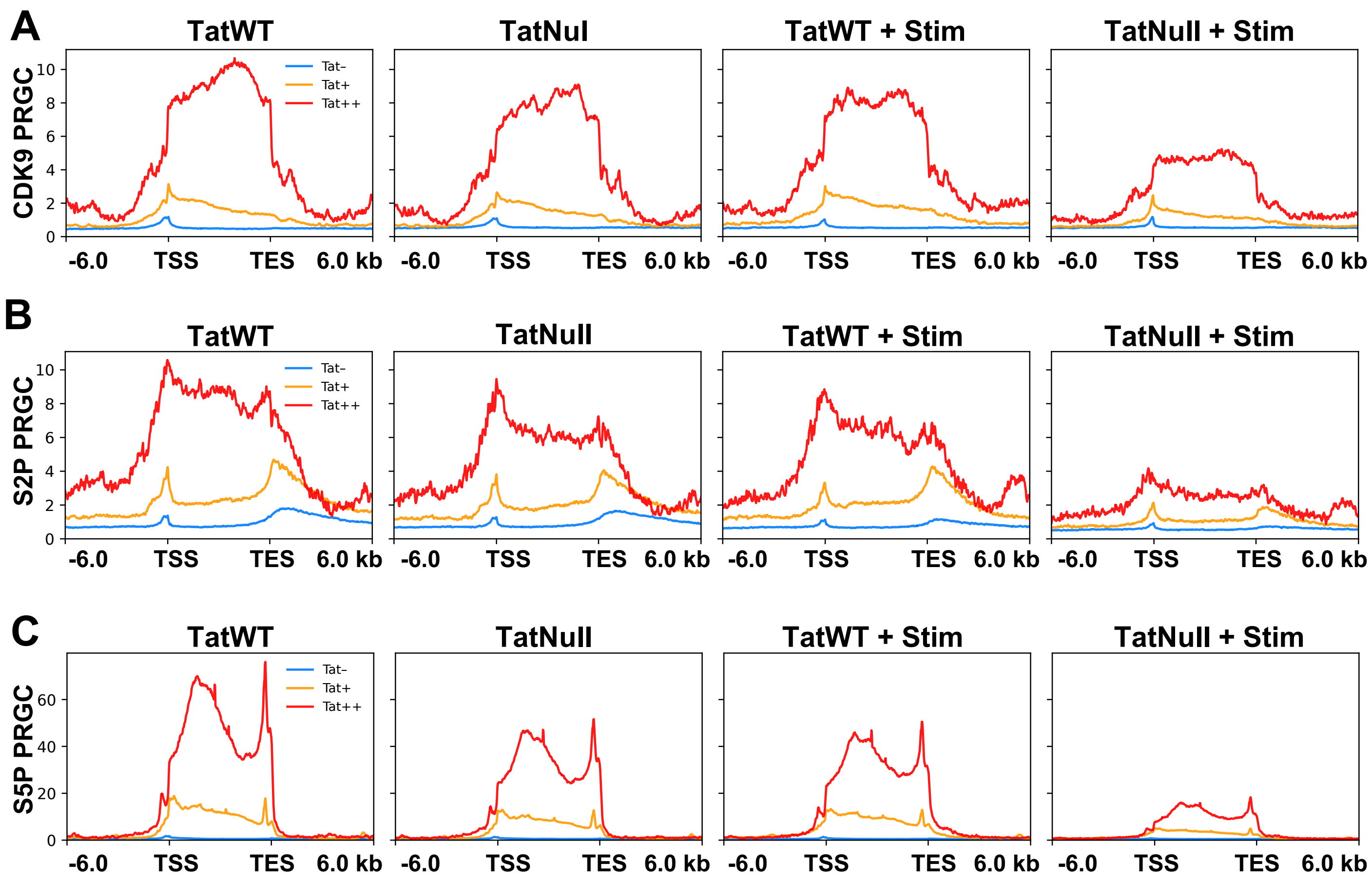
A
B
C
TatWT
TatNul
TatWT + Stim
TatNull + Stim
TatNull
CDK9 PRGC
S2P PRGC
S5P PRGC
Tat–
Tat+
Tat++
-6.0
TSS
TES
6.0 kb
0
2
4
6
8
10
20
40
60

Figure S7

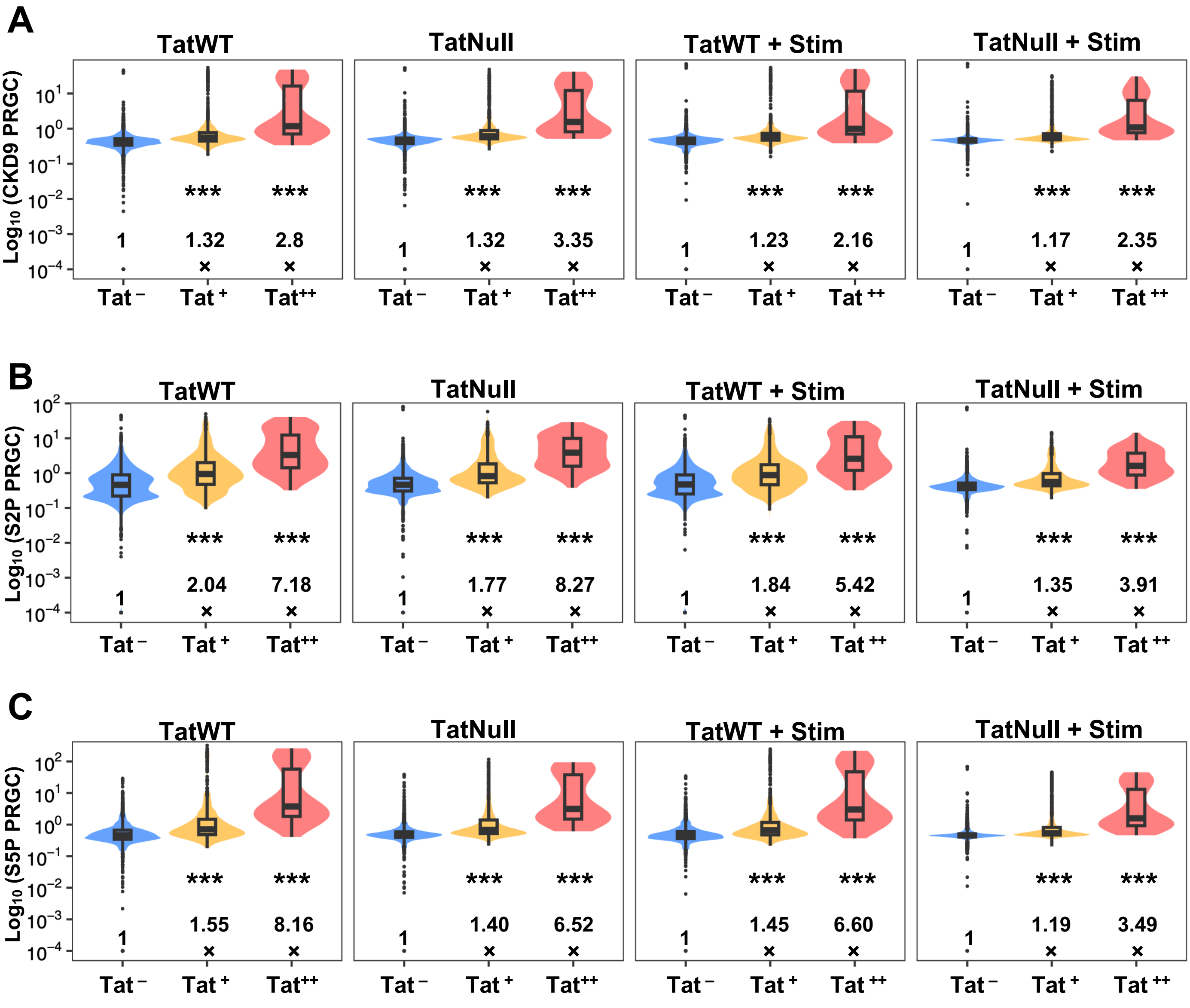

A
B
C
TatWT
TatNull
TatWT + Stim
TatNull + Stim
Log10 (CKD9 PRGC)
Log10 (S2P PRGC)
Log10 (S5P PRGC)
Tat −
Tat +
Tat ++
***
1
1.32
2.8
3.35
1.23
2.16
1.17
2.35
2.04
7.18
1.77
8.27
1.84
5.42
1.35
3.91
1.55
8.16
1.40
6.52
1.45
6.60
1.19
3.49

Figure S8

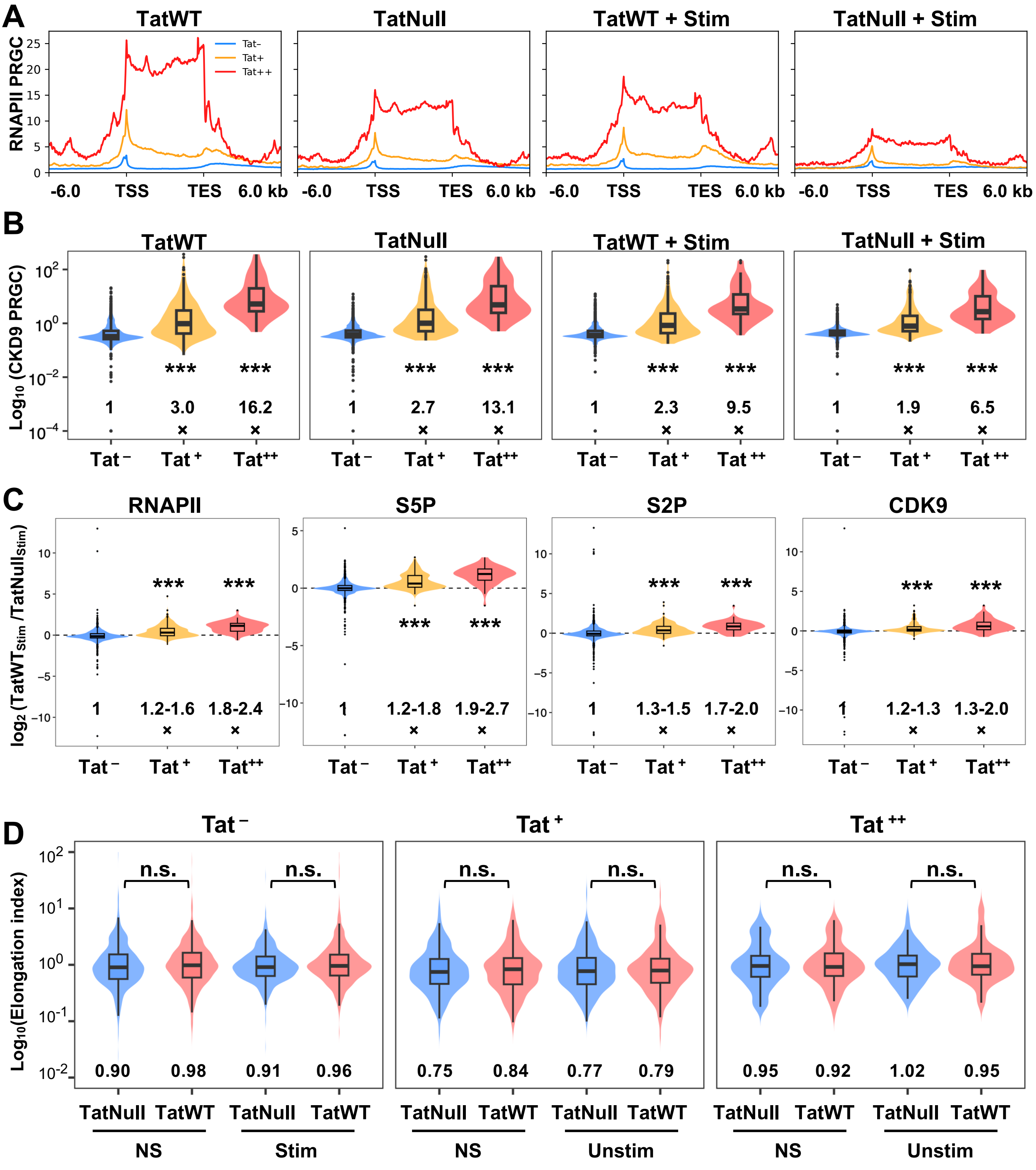
A
TatWT
TatNull
TatWT + Stim
TatNull + Stim
RNAPII PRGC
Tat-
Tat+
Tat++
-6.0
TSS
TES
6.0 kb
B
TatWT
TatNull
TatWT + Stim
TatNull + Stim
Log10 (CKD9 PRGC)
***
1 3.0 16.2
1 2.7 13.1
1 2.3 9.5
1 1.9 6.5
Tat− Tat+ Tat++
C
RNAPII
S5P
S2P
CDK9
log2 (TatWTStim /TatNullStim)
***
1 1.2-1.6 1.8-2.4
1 1.2-1.8 1.9-2.7
1 1.3-1.5 1.7-2.0
1 1.2-1.3 1.3-2.0
Tat− Tat+ Tat++
D
Tat−
Tat+
Tat++
Log10(Elongation index)
n.s.
0.90 0.98 0.91 0.96
0.75 0.84 0.77 0.79
0.95 0.92 1.02 0.95
TatNull TatWT
NS
Stim
Unstim

Figure S9

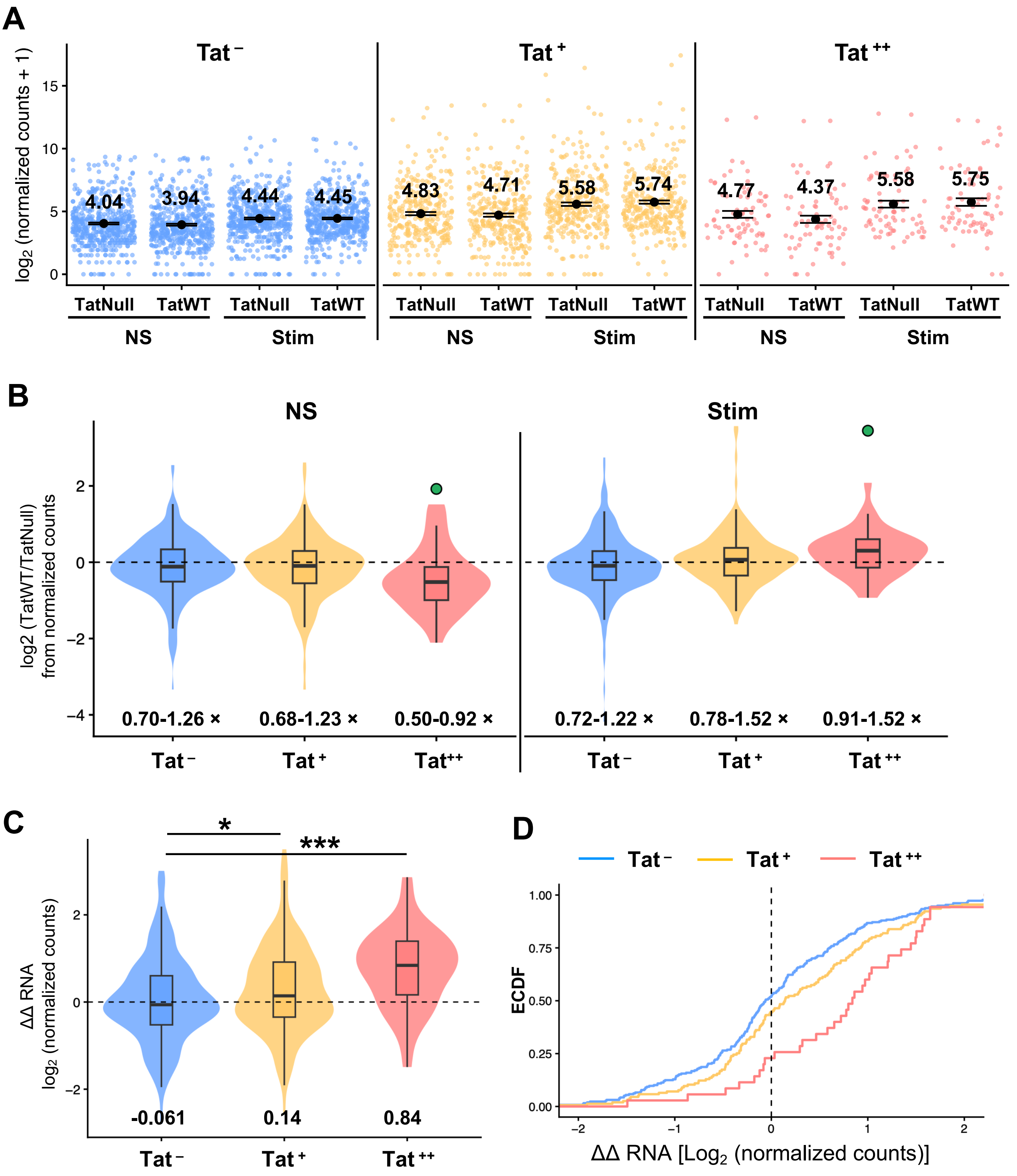
A
Tat⁻
Tat⁺
Tat⁺⁺
log2 (normalized counts + 1)
0
5
10
15
4.04
3.94
4.44
4.45
4.83
4.71
5.58
5.74
4.77
4.37
5.58
5.75
TatNull
TatWT
TatNull
TatWT
NS
Stim
B
NS
Stim
log2 (TatWT/TatNull) from normalized counts
2
0
−2
−4
0.70-1.26 ×
0.68-1.23 ×
0.50-0.92 ×
0.72-1.22 ×
0.78-1.52 ×
0.91-1.52 ×
Tat⁻
Tat⁺
Tat⁺⁺
C
*
***
ΔΔ RNA log2 (normalized counts)
2
0
−2
-0.061
0.14
0.84
Tat⁻
Tat⁺
Tat⁺⁺
D
Tat⁻
Tat⁺
Tat⁺⁺
ECDF
1.00
0.75
0.50
0.25
0.00
−2
−1
0
1
2
ΔΔ RNA [Log2 (normalized counts)]

Figure S10

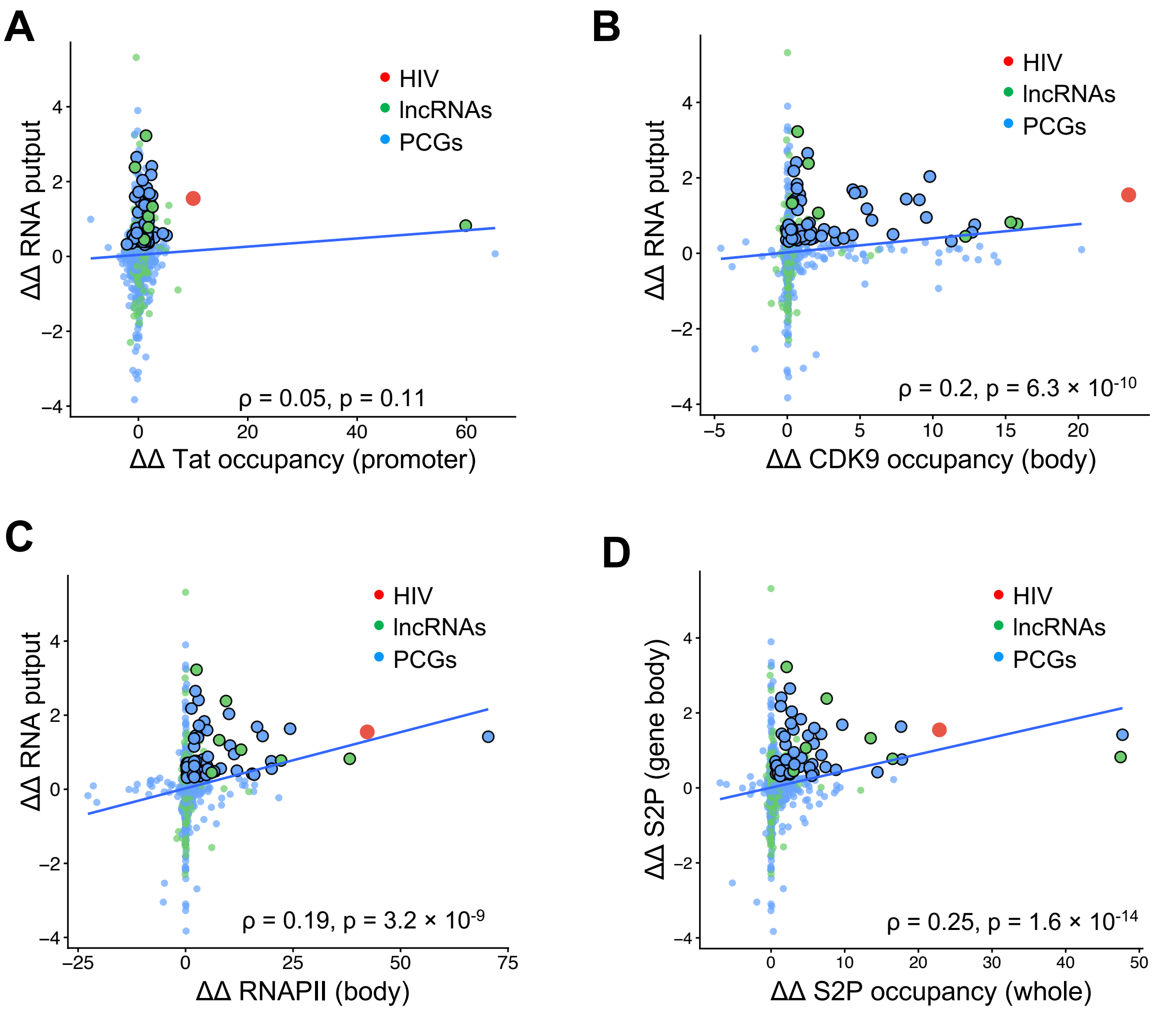

A
HIV
lncRNAs
PCGs
ΔΔ RNA putput
ρ = 0.05, p = 0.11
ΔΔ Tat occupancy (promoter)
B
HIV
lncRNAs
PCGs
ΔΔ RNA putput
ρ = 0.2, p = 6.3 × 10-10
ΔΔ CDK9 occupancy (body)
C
HIV
lncRNAs
PCGs
ΔΔ RNA putput
ρ = 0.19, p = 3.2 × 10-9
ΔΔ RNAPII (body)
D
HIV
lncRNAs
PCGs
ΔΔ S2P (gene body)
ρ = 0.25, p = 1.6 × 10-14
ΔΔ S2P occupancy (whole)

Supplementary Table S1. The Tat ChIP signal of Tat^{++} protein coding genes (PCGs)

Gene_name	TatWT ChIP(RPGC)	TatNull ChIP(RPGC)	log2(TatWT/TatNull)
**GALE**	0.752512093935158	0.37575413497286	1.00192386560686
**ARMH1**	1.13586122217833	0.528773811963125	1.10306248819509
**PTCH2**	0.986894320116573	0.279637329319918	1.81933494545029
**TENT5C**	1.2904926584867	0.601676016881743	1.10086189776981
**H2BC18**	0.922370776321334	0.395599260656284	1.22130499192905
**H3C13**	10.65187765337	3.9259087724218	1.44000897708704
**H2AC18**	11.9507540614227	5.81633840102316	1.03891840964125
**H2AC19**	12.1574575376849	6.02643083511515	1.01246571062671
**H3C15**	10.4755074973556	5.040348423132	1.05542461887059
**H2BC21**	5.33625656040343	2.4800725142958	1.10544541589582
**H2AC20**	16.1093828089324	7.2909917329487	1.14371414268536
**BOLA1**	1.97219342694669	0.513645488323236	1.94095409877375
**H2AW**	3.53307831940038	1.52552983960626	1.2116147935808
**ATP5F1C**	0.626107778076308	0.299718593011893	1.06279993046143
**TSSC4**	0.68647420831788	0.294184870054784	1.22247966451583
**SMPD1**	0.515484917718217	0.216578224150333	1.25103816011004
**SLC25A45**	0.670672383908562	0.261644794650139	1.35799527777723
**RAB1B**	0.618111814343976	0.299761596897352	1.04404979328122
**NPAS4**	0.848637573062006	0.406990250945927	1.06015247341448
**H2AX**	2.38023774560352	0.988652415878268	1.2675695267909
**TPI1**	1.40677294914678	0.582901973116803	1.27106285898594
**IL23A**	0.875106975543826	0.39911483020914	1.13265353354685
**CDK4**	0.904973799608198	0.432744663777564	1.06435825523976
**HCFC1R1**	1.10782584860006	0.441433835402579	1.32746002018945
**THOC6**	0.908995188686721	0.377223114152797	1.26885234175397
**USP7**	1.02652917448373	0.502413705080026	1.03082543964379
**OVCA2**	1.19946869299283	0.290413988366766	2.04620888740521
**TXNDC17**	1.05466689398559	0.478117290922588	1.14134927427361
**EIF5A**	1.25299082980658	0.559635937843428	1.16281391424298
**MPDU1**	0.854448182327518	0.350908309121766	1.28389647045231
**TMEM107**	5.26124123956202	1.97178380758666	1.41590136600812
**RPL19**	1.1580776822998	0.548966065340264	1.07694177231255

**DDX5**	1.12273516284965	0.529088496812843	1.08543525817528
**ARHGDIA**	3.14984184432035	1.55046055146682	1.02258209949523
**MYL12A**	0.989112437825132	0.431976241272516	1.19518068194266
**C19orf25**	0.562903748601828	0.248936078607123	1.17710968622929
**MRPL54**	0.574076807528378	0.259850821017734	1.1435571162401
**SNAPC2**	0.555854209910706	0.262165750746788	1.08422440867118
**STX10**	0.701238820687687	0.335375455138128	1.06412651438175
**SERTAD1**	0.724572043474628	0.349416368694115	1.05217981081344
**JOSD2**	0.595212920588569	0.297284642533053	1.00155847823138
**PTRHD1**	1.11637833627025	0.483894762756002	1.20605911467088
**DUSP2**	0.899076216395052	0.405854266923918	1.14747968957102
**SLC11A1**	0.579506542565351	0.280465440836085	1.04699928298064
**LGALS1**	0.97864018413691	0.433468190324315	1.17485054225217
**POLDIP3**	0.848525521403607	0.336701470565478	1.33348544608171
**TUSC2**	0.665186930893098	0.323161713318984	1.0415012439483
**ABCF3**	0.551577499619433	0.268284481650151	1.0397972372528
**BCL6-AS1**	1.82886700934444	0.757776267295509	1.2711051926302
**H3C1**	8.99991230326375	4.45900860640008	1.01318779616815
**H4C1**	6.65340183830973	2.98624957675365	1.15575715875946
**H3C2**	12.5531320814517	5.87825166362606	1.09458820857426
**H4C3**	10.3673941459185	2.95418108775292	1.81122279838765
**H2AC6**	1.26106153865373	0.52301026243403	1.2697259049541
**H4C4**	14.6590859363452	5.62262106178416	1.3824802648079
**H3C4**	10.4074487657718	3.86979998437832	1.42728531629975
**H3C8**	7.0710304692196	2.98200138348952	1.2456392691991
**H4C8**	1.33901002769719	0.588261923508508	1.18663482693863
**H4C9**	4.69795196142963	1.87715073991596	1.32348699293211
**H2AC13**	11.1647681166116	5.01292062516715	1.1552298335526
**H3C10**	7.32195016175581	3.62165916129862	1.01557697347279
**H2AC14**	7.74336240221472	2.5470961905828	1.6041063404289
**H4C11**	12.9268452367317	6.28515869302431	1.04034903755378
**H4C12**	15.2561788805387	6.27088302912946	1.28265301480242
**H1-5**	15.0510498319695	3.94482113576145	1.93183205781562
**H3C11**	10.5380156002197	3.33053984727518	1.66177497645494

**H4C13**	14.1929100637904	6.57782612044066	1.10948752980567
**TUBB**	1.50926468518597	0.745717516161965	1.01714372542912
**DXO**	1.4717282856342	0.581705932365411	1.3391479196961
**RING1**	0.537322004828265	0.234847273703523	1.19406061776065
**SMIM29**	1.28458387625357	0.524862537548339	1.29128793482082
**CDKN1A**	0.665855137872224	0.325110880654839	1.03427423042877
**MEA1**	0.776027196923748	0.370456859583293	1.06679963152512
**KLHDC3**	0.725547935493455	0.317977336334347	1.19014444287709
**FAM133B**	2.16704434863547	1.01801289262745	1.08997209456709
**CDK6**	2.13079197280232	0.959012592199728	1.15176726014965
**VGF**	0.874523101964335	0.354633621202112	1.30216475929829
**FMC1**	1.21230055837087	0.585889057874402	1.04904673810296
**FASTK**	1.03480334306418	0.503120541159773	1.04037914904754
**LRRC14**	1.02739473869521	0.409604874974654	1.32668368181269
**TMSB4X**	1.52701716117976	0.701973126900433	1.12122745823353

Supplementary Table S2. The Tat ChIP signal of Tat^{++} lncRNAs.

Gene_name	TatWT ChIP(RPGC)	TatNull ChIP(RPGC)	log2(TatWT/TatNull)
**RP1-163M9.8**	7.68945828150957	3.75464426250268	1.03420554335909
**RNU11**	90.2623735966573	43.4273840343679	1.05551964905466
**RP1-39G22.7**	1.14470717506235	0.543096394294427	1.07569700675762
**RP11-386I14.4**	25.0677455920352	8.47626760017359	1.56433110383887
**CH17-3B23.3**	9.038257607327	3.60566403625942	1.32577945909288
**CH17-373J23.1**	63.2441473089653	27.8054428567804	1.18556463303688
**CH17-353B19.2**	10.4950268245457	3.51705422977651	1.57726609603368
**LINC01719**	1.6853694882351	0.605859049415202	1.47600928604446
**CH17-408M7.2**	23.4345397134421	10.388300231544	1.17367676630755
**RP11-196G18.23**	2.88440625710078	1.34931944274439	1.09604187015092
**RP11-574F21.2**	1.84174280908306	0.523396396366715	1.81509374024612
**RP11-44D15.7**	1.92032737065526	0.775006279207796	1.30907126277712
**RP11-326C3.10**	0.88794265017597	0.212713996751593	2.06154637533842
**RP11-286N22.10**	0.761608230454869	0.311129052230357	1.2915332113991
**RP11-640G3.1**	1.02393483805585	0.510751133441387	1.00343008560064
**U47924.31**	14.618313341219	6.80632552996264	1.10282869103062
**RP11-823E8.3**	1.26449305856973	0.419701773031232	1.59112035783743
**ATP2B1-AS1**	0.941434314930706	0.429925727403111	1.13077117049691
**SBNO1-AS1**	1.71235023559679	0.682036977629521	1.3280546765378
**RP11-290D2.6**	1.42828221633445	0.629171007481204	1.18275569152259
**RPPH1**	14.5028105357598	4.44421033911571	1.70633328117953
**LINC02332**	19.9260426756549	7.16883785388571	1.474843923126
**AE000662.92**	2.38799571573386	0.942049798963466	1.34192408977865
**RP11-109N23.4**	1.9680828295803	0.747764716205942	1.39613344018838
**CTD-2376I20.1**	1.26859710957172	0.614413907548227	1.04594997171797
**POLG-DT**	3.06101258301971	1.1732346708437	1.38351660641921
**SNHG9**	1.10008377366836	0.181265703779383	2.60142885247013
**RP11-333E1.2**	0.552240898940554	0.233441759030331	1.24223151076084
**AC113189.5**	2.01386323516458	0.692626176102792	1.5398155274873
**LINC00910**	1.18819665019549	0.551121405413617	1.10833015553099
**AC142472.6**	2.58508030139873	1.25127566880977	1.04680883639098
**PICART1**	0.80309698048581	0.388224963593452	1.04867940666578

**RP5-1171I10.5**	5.16094728161739	2.04513609328637	1.33543861757997
**RP11-758H9.2**	1.68083735772131	0.669028710580324	1.32903880538027
**RP11-498C9.3**	7.9041957706213	3.07752259706075	1.36084894378136
**AC114271.2**	0.868272225584714	0.259096117999433	1.74465612885505
**CTC-250I14.6**	1.75841254383352	0.773809209850784	1.1842227342081
**AC010504.2**	0.834678658338222	0.391367434049435	1.09269521234594
**AC074212.5**	0.700142629009062	0.286327746561066	1.28997839013007
**DM1-AS**	1.16003950557062	0.333048874261046	1.80036503900467
**CTD-2619J13.23**	2.19492940622903	0.992346887192108	1.14525731783892
**LINC01814**	0.718205364217506	0.301938060061792	1.25014103535552
**CLASP1-AS1**	7.9688481000071	3.8953242785772	1.03262757285813
**LINC00471**	0.714661955197161	0.291355613535247	1.29447694866473
**LINC01431**	0.790555093606209	0.264014554352488	1.58224490430318
**RNU12**	27.0577788981329	12.6971010149243	1.0915442164323
**ARF4-AS1**	1.12936808590145	0.470546543479562	1.26310464287332
**RP11-573D15.9**	1.93874035298824	0.694446767965953	1.48118225381995
**STIM2-AS1**	2.16315564250139	0.65452725956489	1.72461075252187
**RP11-629B11.5**	1.01088111847239	0.501768586780202	1.01051783673347
**XXbac-BPG299F13.17**	1.83248467122778	0.582650799117851	1.65309604624339
**XXbac-BPG181M17.6**	3.9081676921917	0.991074981798237	1.97942516876396
**CTB-85C5.1**	9.83732598941754	2.19278280081752	2.16550278253247
**GASAL1**	1.75830998765704	0.735885756292518	1.25663458270525
